# Inadequate calcium and vitamin D intake and osteoporosis risk in older Americans living in poverty with food insecurities

**DOI:** 10.1371/journal.pone.0235042

**Published:** 2020-07-08

**Authors:** Keri Marshall, Lynn Teo, Christopher Shanahan, LeeCole Legette, Susan Hazels Mitmesser

**Affiliations:** 1 Science & Technology, Pharmavite LLC, West Hills, California, United States of America; 2 Teo Research Consulting, Silver Spring, Maryland, United States of America; 3 Frost and Sullivan, Santa Clara, California, United States of America; University of Massachusetts Boston, UNITED STATES

## Abstract

Poverty may be a barrier to acquiring adequate nutrient levels for the prevention of osteoporosis. Age and nutritional intake are major factors that contribute to osteoporosis prevalence. This study examined the relationship between markers of poverty with calcium / vitamin D intake and osteoporosis. A cross-sectional analysis of the United States population was performed using National Health and Nutrition Examination Survey (NHANES) data from 2007–2010 and 2013–2014 for older US adults (n = 3,901 participants, 50 years old and older). Odds of inadequate calcium / vitamin D intake and dietary supplement use and risk of probable osteoporosis were calculated in order to determine the relative difference and possible associations between household income, the Family Monthly Poverty Level Index, food security, and participation in the Supplemental Nutrition Assistance Program (SNAP). A sub-analysis of ethnic disparities and biological sex was also performed. In general, women age 50 and older consistently have inadequate calcium intake, regardless of economic level including poverty. While inadequate calcium intake has a larger prevalence among women, markers of poverty increased the risk of inadequate calcium intake in all men and risk of osteoporosis among some subgroups, with the exception of SNAP program participation. Over one fourth of Non-Hispanic black men in the US are below the poverty line. Approximately half of this population has inadequate calcium (58.9%) and vitamin D (46.7%) intake. Typically, osteoporosis risk is relatively low for Non-Hispanic Black males, however considering poverty status, risk is significantly increased (Relative Risk Ratio [RR]: 2.057 ± 0.012) for those with low income suggesting that calcium and vitamin D supplementation may be of benefit for this population.

## Introduction

Rising numbers of people consuming nutrient poor diets, coupled with aging, sedentary lifestyle and low income, can contribute to a number of chronic diseases. Nutritional intake along with health disparities, further delineated by socio-economic status, can contribute to a higher prevalence of disease among low-income populations [1]. Poverty-stricken populations may lack resources to acquire adequate levels of calcium and vitamin D for the prevention of osteoporosis. This study analyzes the relationship between markers of poverty with calcium and vitamin D intake as well as incidences of osteoporosis in older Americans (≥ 50 years of age).

Osteoporosis is a prevalent and debilitating metabolic bone disease characterized by changes in skeletal structure and reduction in bone mass, leading to increased bone fragility and risk of fracture [2]. Arriving at a diagnosis of osteoporosis is a multifactorial process which includes taking a case history, physical examination, and diagnostic imaging. Common imaging testing consists of a dual-X-ray absorptiometry (DXA) scan to determine a measurement of bone mineral density (BMD) in the femoral neck and lumbar spine of the patient [2, 3]. In this context, osteoporosis is defined as a T-score equal to or less than − 2.5 standard deviations from the BMD of a healthy 25-year old of the same gender at their peak bone mass. Osteopenia, or low bone mass, is defined as a T-score of between − 1.0 and − 2.5 SD [2]. Incidence of fractures attributed to osteoporosis increase with age and frequently occur in the spine, hip or wrist [2, 4].

It has been estimated in the US population age 50 and older, that 10.3% or 10.2 million suffer from osteoporosis, and 80% of these affected cases are females. In addition, there are potentially 43.4 million people, or 44% of the population with osteopenia [3]. Over two million osteoporosis-related fractures occur annually, leading to more than 19 billion dollars in health care costs in the US [4, 5].

Bone is a living tissue with nutrients serving an integral role in its structural integrity [6]. Inadequate nutrition is a major risk factor for osteoporosis. Calcium is the mineral with the highest prevalence in the human body and is necessary for normal bone development [7]. Adequate levels of calcium intake during youth is essential for establishing peak bone mass [8]. Inadequate calcium levels during adulthood are associated with increased risk for low bone density, osteoporosis, fractures and falls [8]. Additionally, calcium is necessary for the function of nerves and muscles, circulation of blood through blood vessels, and the release of hormones and enzymes necessary for normal body function [9]. According to the Food and Nutrition Board of the National Academies of Medicine (formerly known as Institute of Medicine), the recommended daily amount of calcium for adults is 1000 mg for men 51–70 years old, 1200 mg for women 51–70 years, and 1200 mg for all adults 71 years and older [9]. Dietary sources of calcium include dairy and soy products, certain dark leafy greens, nuts, legumes and calcium-fortified foods [10].

Vitamin D is a fat-soluble vitamin that helps with the absorption of calcium. Adequate levels of vitamin D are essential for proper function of nerves, muscles and the immune system. The recommended daily amount of vitamin D is 15 mcg (600 IU) for adults up to 70 years old, and 20 mcg (800 IU) for individuals 71 years and older [9]. Dietary sources of vitamin D include fatty fish (i.e. salmon, tuna and mackerel), beef liver, egg yolks and vitamin D-fortified foods. Exposure to direct sunlight can also increase vitamin D levels [9].

Intake of calcium and vitamin D supplements can also help to meet daily recommended amounts. While intake of calcium and vitamin D rich foods decreases with age, evidence shows that supplementation can help individuals meet recommended intake levels [7]. A recent meta-analysis of eight randomized controlled trials reported that supplementation of calcium and vitamin D significantly reduced the risk of fractures by 15% and reduced the risk of hip fractures specifically by 30% [11].

The 2018 United States Census Bureau reported that 11.8% of the US population lived at or below the poverty line. Low-income populations in the US have been shown to be at risk for inadequate calcium and vitamin D intake [7]. In this investigation, the relationship between nutrient intake and prevalence of osteoporosis and a number of poverty indicators were examined. Annual household income, the Family Month Level Poverty (FMLP) Index Category, self-reported food insecurity, and Supplemental Nutrition Assistance Program (SNAP; formally the Food Stamp Program) participation were utilized as poverty indicators. These indicators were evaluated to determine their influence and effect on the intake of calcium and vitamin D and the occurrence of osteoporosis in older Americans.

An annual household income of <$20,000 was chosen in accordance to the 2019 Poverty Guidelines issued by the US Health and Human Services (USHHS). USHHS notes the poverty level for a family of three is an annual household income of $21,330 within the contiguous US. Levels are higher for Alaska and Hawaii [12]. The FMLP Index is a ratio of monthly income to HHS 2017 poverty guidelines. NHANES categorizes respondents into three separate FMLP Index Categories (≤1.30, 1.30 to 1.85, and >1.85) in accordance to commonly used percentages of the poverty guidelines of 130% and 185%.

According to the United States Department of Agriculture (USDA), 11.1% of Americans reported household food insecurity in 2018 where one or more members of their household had to reduce or change their eating patterns due to lack of funds or resources to obtain food [13]. A recent study analyzing the intake of several micronutrients by food security status found that adults living in food insecure households typically did not meet dietary reference intakes for several micronutrients, including vitamin D [14].

Administered by the Food and Nutrition Service (FNS) of the USDA, SNAP is the US government’s main effort to reduce food insecurity by providing nutritional assistance to low income households and individuals [15]. In 2017, 84% of eligible individuals (45 million) participated in this program [16]. In general, the benefit amount is based on the maximum household benefit minus the expected contribution (30% of net income). For example, the current maximum monthly benefit for a household of three is $509 and the average monthly benefit for the fiscal year 2020 is estimated to be $378. Households who live at or 130% below the poverty level are eligible for SNAP, although households with older adults (≥ 60 years of age) are able to participate at a slightly higher income bracket. While the SNAP program has been shown to decrease levels of food insecurity [17], there is evidence to suggest the quality of food consumed by SNAP participants does not meet the standards for a healthy diet [1].

The purpose of our study was to analyze the relationship between markers of poverty with adequate intake of calcium and vitamin D, and assess these markers against the incidence of osteoporosis in older Americans (≥ 50 years of age). Data from the USHSS’s Center for Disease Control and Prevention’s (CDC) biennial National Health and Nutrition Examination Survey (NHANES) was utilized to determine the relative risk of inadequate nutrient intake (inadequate calcium and vitamin D food intake and/or supplementation). This study related several poverty indicators (described above) to the relative risk of osteoporosis, and explored the combined effect of poverty markers and nutrient intake on the relative risk of osteoporosis. Additional sub-analyses were performed by biological sex and ethnicity.

## Materials and methods

NHANES provides a rich source of patient information that can be used to quantify the risk of nutrient intake inadequacy and probable osteoporosis across various poverty indicators, gender, and ethnicities because it continuously tracks the health and nutritional status of Americans over time. Specifically, NHANES provides the prospect to identify and explore the connection between socio-economic indicators, such as poverty, ethnicity, health outcomes and food and nutrient intake levels. The type of information available per subject observation includes demographics, responses to dietary intake questionnaires, clinical examination data, laboratory data, and other lifestyle questionnaire data. The NHANES study protocol was approved by the Research Ethics Review Board of the National Center for Health Statistics and all participants provided written informed consent. Three editions of NHANES spanning from 2007 to 2010 and 2013 to 2014 were used to build a complete dataset of 3,901 observations for all older Americans age of 50 and older [18–20]. It should be noted that the NHANES 2011–2012 examination data for bone density measurements is not publicly available and/or was not measured. Therefore, we compiled all three datasets available that have measured BMD over the past 10 years to build up a sufficiently large and representative sample. This dataset was used to measure the expected burden of osteoporosis in the United States including complete measurements of femur and spine BMD per subject observation along with associated socio-economic demographic indicators, body measurements and data on health of diet, general health, and nutrient intake including calcium and vitamin D.

For each observation, gender (biological sex), age and ethnicity were obtained from the demographic variables available in the NHANES “DEMO” file. From the dietary interview statistics available in NHANES, total dietary (food-based) intake of calcium and vitamin D for both the first day (DR1TOT) and second day (DR2TOT) and the 30 day dietary supplement use (DSQTOT) of any type of supplement, calcium supplements, and vitamin D supplements were obtained in order to calculate total average intake per observation. Nutrient intake calculations were compared to the Institute of Medicine’s recommended dietary reference intake for calcium and vitamin D. Inadequate nutrient intake was defined as reported intake less than recommended intake levels. Specifically, an individual was deemed to have inadequate calcium and/or vitamin D intake if their average daily intake from both food and supplements was less than 800 to 1,100 milligrams and/or less than 10 micrograms (400 IU) respectively, depending on their age and gender. Furthermore, use and non-use of any type of dietary supplement and use and non-use of calcium and vitamin D supplements was coded based on each survey participant’s reported behavior.

In order to quantify the impact of poverty and food security on the risk of nutrient intake inadequacy and probable osteoporosis across gender and ethnicity, various poverty indicators were extracted from NHANES including total household income per observation (INDHHIN2) and each observation’s reported FMPL index (INDFMMPI). Individuals who had a FMPL Index score of ≤ 1.30 was considered below the poverty line and aligned with qualification score for SNAP. In addition, NHANES’ categorization of Household Food Security (FSDHH) and current household participation in SNAP (FSQ012) were also used as poverty indicators. All information was matched and organized by the respondent sequence using Microsoft Access to create a complete dataset. See Table 1 for a description of the summary statistics for each cohort explored in this study.

**Table 1:** Estimated population and sample sizes of key cohorts, United States, age 50 and older.

Demographic Parameter		Population	Inadequate Calcium Intake	Inadequate Vitamin D Intake	Calcium & Vitamin D Supplement Users
**Female Cohort**	**Total Population**	70.66 (100%) [1992]	51.14 (72.4%) [1476]	24.07 (34.1%) [760]	17.01 (24.1%) [440]
	***Mexican American***	[332]	[241]	[160]	[71]
	***Other Hispanic***	[218]	[170]	[97]	[39]
	***Total Hispanic***	7.47 (10.6%) [550]	5.58 (7.9%) [411]	3.49 (4.9%) [257]	1.49 (2.1%) [110]
	***Non-Hispanic Black***	7.87 (11.1%) [344]	6.56 (9.3%) [287]	3.98 (5.6%) [174]	1.07 (1.5%) [47]
	***Non-Hispanic White***	50.55 (71.5%) [951]	35.29 (49.9%) [664]	15.2 (21.5%) [286]	13.5 (19.1%) [254]
	***All Other People***	4.77 (6.8%) [147]	3.7 (5.2%) [114]	1.4 (2%) [43]	0.94 (1.3%) [29]
	**HH income below $20,000 per year**	17.81 (25.2%) [524]	13.24 (18.7%) [398]	7.01 (9.9%) [221]	4.13 (5.8%) [112]
	**Monthly poverty level index less than or equal to 1.3**	18.9 (26.7%) [602]	14.48 (20.5%) [464]	8.37 (11.8%) [279]	3.78 (5.3%) [115]
	**Food Insecure**	8.43 (11.9%) [299]	6.42 (9.1%) [228]	4.11 (5.8%) [149]	1.73 (2.4%) [57]
	**SNAP Participants**	8.68 (12.3%) [287]	6.71 (9.5%) [222]	4.15 (5.9%) [139]	1.66 (2.3%) [55]
**Male Cohort**	**Total Population**	62.02 (100%) [1909]	32 (51.6%) [1035]	24.63 (39.7%) [814]	23.53 (37.9%) [685]
	***Mexican American***	[316]	[168]	[143]	[120]
	***Other Hispanic***	[195]	[120]	[95]	[57]
	***Total Hispanic***	6.67 (10.8%) [511]	3.76 (6.1%) [288]	3.11 (5%) [238]	2.31 (3.7%) [177]
	***Non-Hispanic Black***	6.14 (9.9%) [363]	3.91 (6.3%) [231]	3.23 (5.2%) [191]	1.71 (2.8%) [101]
	***Non-Hispanic White***	45.29 (73%) [899]	22.12 (35.7%) [439]	16.83 (27.1%) [334]	18.19 (29.3%) [361]
	***All Other People***	3.91 (6.3%) [136]	2.21 (3.6%) [77]	1.47 (2.4%) [51]	1.32 (2.1%) [46]
	**HH income below $20,000 per year**	12.74 (20.5%) [415]	7.51 (12.1%) [258]	5.95 (9.6%) [210]	4.26 (6.9%) [127]
	**Monthly poverty level index less than or equal to 1.3**	15.25 (24.6%) [533]	8.87 (14.3%) [322]	7.55 (12.2%) [268]	4.92 (7.9%) [166]
	**Food Insecure**	6.46 (10.4%) [265]	3.77 (6.1%) [163]	3.36 (5.4%) [140]	2.03 (3.3%) [80]
	**SNAP Participants**	7.17 (11.6%) [266]	3.68 (5.9%) [146]	3.14 (5.1%) [122]	2.88 (4.6%) [101]
**All**	**Total Population**	132.68 (100%) [3901]	83.14 (62.7%) [2511]	48.7 (36.7%) [1574]	40.54 (30.6%) [1125]
	***Mexican American***	[648]	[409]	[303]	[191]
	***Other Hispanic***	[413]	[290]	[192]	[96]
	***Total Hispanic***	14.14 (10.7%) [1061]	9.34 (7%) [699]	6.6 (5%) [495]	3.8 (2.9%) [287]
	***Non-Hispanic Black***	14.01 (10.6%) [707]	10.47 (7.9%) [518]	7.21 (5.4%) [365]	2.78 (2.1%) [148]
	***Non-Hispanic White***	95.84 (72.2%) [1850]	57.41 (43.3%) [1103]	32.03 (24.1%) [620]	31.69 (23.9%) [615]
	***All Other People***	8.69 (6.5%) [283]	5.92 (4.5%) [191]	2.86 (2.2%) [94]	2.26 (1.7%) [75]
	**HH income below $20,000 per year**	30.55 (23%) [939]	20.75 (15.6%) [656]	12.95 (9.8%) [431]	8.39 (6.3%) [239]
	**Monthly poverty level index less than or equal to 1.3**	34.15 (25.7%) [1135]	23.35 (17.6%) [786]	15.92 (12%) [547]	8.71 (6.6%) [281]
	**Food Insecure**	14.89 (11.2%) [564]	10.19 (7.7%) [391]	7.47 (5.6%) [289]	3.77 (2.8%) [137]
	**SNAP Participants**	15.85 (11.9%) [553]	10.39 (7.8%) [368]	7.29 (5.5%) [261]	4.54 (3.4%) [156]

Total population in millions of people. Share of estimated population in parentheses. Sample Size in brackets. Source: Centers for Disease Control and Prevention’s (CDC) and author analysis

From the examination data available in NHANES, BMD (g/cm^2^) using DXA measures for both the femur (DXXFEM) and the spine (DXXSPN) was obtained in order to calculate probable osteoporosis. In this study, the risk of osteoporosis was calculated by counting the number of individuals per cohort that had an observed BMD measurement at the femur or the spine defined as osteoporotic detailed earlier. See Table 2 for the calculated risk of probable osteoporosis by major cohorts including ethnicity, biological sex, nutrient intake status, and poverty status.

**Table 2:** Risk of osteoporosis, United States, by major population cohorts, %, age 50 and older.

Demographic Parameter		Population	Inadequate Calcium Intake	Inadequate Vitamin D Intake	Calcium & Vitamin D Supplement Users
**Female Cohort**	**Total Population**	16.2 (0.01)	16.3 (0.01)	16.6 (0.02)	16.4 (0.03)
	***Mexican American***	19.3 (0.02)	19.5 (0.03)	22.5 (0.05)	22.5 (0.09)
	***Other Hispanic***	21.6 (0.03)	24.1 (0.04)	20.6 (0.07)	15.4 (0.11)
	***Non-Hispanic Black***	8.4 (0.02)	8.7 (0.02)	6.9 (0.01)	4.3 (0.06)
	***Non-Hispanic White***	15 (0.01)	15.1 (0.01)	17.8 (0.03)	14.6 (0.04)
	***All Other People***	27.2 (0.05)	24.6 (0.05)	16.3 (0.09)	37.9 (0.17)
	**HH income below $20,000 per year**	21.6 (0.04)	21.1 (0.04)	19.5 (0.05)	24.1 (0.08)
	**Monthly poverty level index less than or equal to 1.3**	17.8 (0.03)	16.4 (0.04)	15.4 (0.04)	24.3 (0.08)
	**Food Insecure**	17.4 (0.04)	15.8 (0.05)	13.4 (0.04)	26.3 (0.11)
	**SNAP Participants**	15.3 (0.04)	16.2 (0.04)	16.5 (0.05)	9.1 (0.03)
**Male Cohort**	**Total Population**	4.6 (0.03)	5.2 (0.04)	4.3 (0.03)	3.9 (0.02)
	***Mexican American***	3.8 (0.12)	4.2 (0.14)	4.9 (0.05)	2.5 (0.13)
	***Other Hispanic***	4.6 (0.11)	2.5 (0.39)	--	10.5 (0.07)
	***Non-Hispanic Black***	1.9 (0.28)	2.6 (0.24)	2.6 (0.15)	--
	***Non-Hispanic White***	5.2 (0.04)	7.3 (0.03)	5.1 (0.03)	3.3 (0.04)
	***All Other People***	8.8 (0.03)	7.8 (0.06)	9.8 (0.04)	10.9 (0.08)
	**HH income below $20,000 per year**	6.3 (0.02)	6.2 (0.02)	6.2 (0.01)	6.3 (0.02)
	**Monthly poverty level index less than or equal to 1.3**	6.8 (0.02)	8.1 (0.03)	7.5 (0.03)	4.2 (0.01)
	**Food Insecure**	4.2 (0.01)	3.7 (0.02)	3.6 (0.1)	3.8 (0.06)
	**SNAP Participants**	7.9 (0.01)	7.5 (0.02)	9 (0.01)	6.9 (0)
**All**	**Total Population**	10.5 (0.01)	11.7 (0.01)	10.2 (0.01)	8.8 (0.01)
	***Mexican American***	11.7 (0.02)	13.2 (0.03)	14.2 (0.03)	9.9 (0.03)
	***Other Hispanic***	13.6 (0.03)	15.2 (0.04)	10.9 (0.02)	12.5 (0.06)
	***Non-Hispanic Black***	5.1 (0.00)	6 (0.01)	4.7 (0.03)	2 (0.17)
	***Non-Hispanic White***	10.3 (0.01)	12 (0.02)	11 (0.01)	8 (0.01)
	***All Other People***	18.4 (0.04)	17.8 (0.05)	12.8 (0.04)	21.3 (0.09)
	**HH income below $20,000 per year**	14.8 (0.02)	15.2 (0.03)	13 (0.03)	14.6 (0.04)
	**Monthly poverty level index less than or equal to 1.3**	12.6 (0.02)	13 (0.03)	11.5 (0.03)	12.5 (0.04)
	**Food Insecure**	11.2 (0.02)	10.7 (0.03)	8.7 (0.01)	13.1 (0.05)
	**SNAP Participants**	11.8 (0.02)	12.8 (0.03)	13 (0.03)	7.7 (0.01)

Standard Errors are in parentheses. Source: Centers for Disease Control and Prevention’s (CDC) and author analysis

After determining risk estimates for nutrient intake inadequacy and probable osteoporosis across various factors (poverty indicators, gender, and ethnicities), increased odds of an individual having inadequate nutrient intake and their relative risk of probable osteoporosis given an individual’s current poverty level and ethnicity was calculated to assess whether any of the risk factors explored in this study could be used as guidance for predicting at-risk populations. In some cases, it was not possible to calculate with statistical confidence the relative risk of nutrient intake inadequacy and probable osteoporosis for some cohorts due to the low number of observed cases in the sample, particularly among the male cohort which was expected due to the low risk of osteoporosis for men.

The following relationships were explored by ethnicity and biological sex:

- Living below the poverty line
  ○ The odds of inadequate calcium or vitamin D intake for participants living at or below the poverty line versus those who live above the poverty line
  ○ The odds of calcium and vitamin D supplement use for participants living at or below the poverty line versus those who live above the poverty line
  ○ The relative risk of osteoporosis among those with inadequate calcium or vitamin D intake for participants living at or below the poverty line versus those who live above the poverty line
  ○ The relative risk of osteoporosis among calcium and vitamin D supplement users for participants living at or below the poverty line versus those who live above the poverty line
- Living in households earning less than $20,000 per year
  ○ The odds of inadequate calcium or vitamin D intake for participants living in households earning less than or equal to $20,000 per year versus those who live in households earning more than $20,000 per year
  ○ The odds of calcium and vitamin D supplement use for participants living in households earning less than or equal to $20,000 per year versus those who live in households earning more than $20,000 per year
  ○ The relative risk of osteoporosis among those with inadequate calcium or vitamin D intake for participants living in households earning less than or equal to $20,000 per year versus those who live in households earning more than $20,000 per year
  ○ The relative risk of osteoporosis among calcium and vitamin D supplement users for participants living in households earning less than or equal to $20,000 per year versus those who live in households earning more than $20,000 per year
- Self-reported food insecurity
  ○ The odds of inadequate calcium or vitamin D intake for participants living food insecure versus food secure
  ○ The odds of calcium and vitamin D supplement use for participants living food insecure versus food secure
  ○ The relative risk of osteoporosis among those with inadequate calcium or vitamin D intake for participants living food insecure versus food secure
  ○ The relative risk of osteoporosis among calcium and vitamin D supplement users between participants living food insecure versus food secure
- SNAP participation
  ○ The odds of inadequate calcium or vitamin D intake for SNAP participants and non-SNAP participants
  ○ The odds of calcium and vitamin D supplement use for SNAP participants and non-SNAP participants
  ○ The relative risk of osteoporosis among those with inadequate calcium or vitamin D intake for SNAP participants and non-SNAP participants
  ○ The relative risk of osteoporosis among calcium and vitamin D supplement users for SNAP participants and non-SNAP participants

## Results

Close to 14 million Americans age 50 and older are expected to suffer from probable osteoporosis in 2019 given current total U.S. population estimates. This number represents over 10% of total Americans in this age group, and within this figure a greater percentage of women are affected overall, 16.2% compared to 4.6% of males. From a nutrient intake perspective, over 60% of this cohort have inadequate calcium intake (72.4% of females; 51.4% of males) while 36.7% (24.1% of females; 39.7% of males) of this cohort have inadequate vitamin D intake. Our analysis demonstrates that only 30% of Americans age 50 or older supplement with calcium and vitamin D (24.1% of females and 37.8% of males).

The results of our study are broken down by ethnic groups in order to control for non-poverty and non-nutrient intake factors that may drive differences in observed cases of probable osteoporosis. For example, this analysis shows that that Non-Hispanic Blacks age 50 and older are the most affected by inadequate nutrient intake with almost three-quarters of this population having inadequate calcium intake (83.4% of females; 63.7% of males) and just over half demonstrate inadequate vitamin D intake (50.6% of females; 52.6% of males). Less than 20% of the Non-Hispanic Black population use calcium and vitamin D supplements, making them the population least likely to supplement in all cohorts examined. Despite these nutrient gaps, only 5% of total Non-Hispanic Blacks were affected by osteoporosis (8.4% of females; 1.9% of males). Conversely, the highest prevalence of osteoporosis appears in individuals of Other Races with 18.4% affected (27.2% of females, 8.8% of males). Other Hispanic females age 50 and older were affected by osteoporosis at a rate of 21.6%. Non-Hispanic Whites comprise 72% of Americans age 50 and older and the prevalence of osteoporosis among this cohort is similar to the prevalence of osteoporosis in the general population for both females and males. This suggests that other factors besides nutrient intake and poverty status impact the relative risk of probable osteoporosis across ethnicities, some of which could likely to be related to genetic and general health profiles. In addition, it should be noted that there were differences in the sampling rates across ethnicities in the observed dataset as shown in Table 1. This can cause some uncertainty to be introduced in the results, however, all ethnicity cohorts sampled in the NHANES survey except for Non-Hispanic Whites were sampled at rates greater than their actual population share as reported by the US Census. Thus, the relative differences in observed cases of probable osteoporosis across ethnicities is likely close to actual population rates.

### Annual household income below 20,000

An estimated 23% of Americans (25.2% females, 20.1% males) age 50 and older (close to 30 million) belong to households with an annual income below $20,000. Over 28% of Non-Hispanic Blacks age 50 and older belong to this income bracket—the highest across ethnicities. Among this income bracket, 68% (74.3% of females; 58.9% of males) have inadequate calcium intake; 43% (39.4% of females; 46.7% of males) have inadequate vitamin D intake; and only 27.5% (23.2% of females; 34.2% of males) use calcium and vitamin D supplements (See Table 1). Over 14% (21.6% of females; 6.3% of males) of individuals within this income bracket have osteoporosis. See Table 3 for the full analysis of influence of annual household income on the relative risk of calcium and vitamin D intake and osteoporosis with analysis by ethnicity and gender.

**Table 3:** Influence of HH income on RR of nutrient intake & osteoporosis with analysis by ethnicity and gender, age 50 and older.

Test		Population	Calcium Intake Inadequate, Poverty versus Non-poverty	Vitamin D Intake Inadequate, Poverty versus Non-poverty	Calcium & Vitamin D Supplement Users, Poverty versus Non-poverty	RR of Osteoporosis, Poverty versus Non-poverty
**Female Cohort**	**RR of Osteoporosis given HH Income below $20,000**	**Total Population**	1.449 ± 0.014✝	1.264 ± 0.018✝	1.442 ± 0.013✝	1.507 ± 0.013✝
		***Mexican American***	1.64 ± 0.04✝	1.983 ± 0.058✝	1.646 ± 0.036✝	1.521 ± 0.033✝
		***Other Hispanic***	1.147 ± 0.052✝	0.91 ± 0.059✝	1.232 ± 0.049✝	1.356 ± 0.047✝
		***Non-Hispanic Black***	1.157 ± 0.016✝	0.684 ± 0.007✝	1.016 ± 0.013✝	0.904 ± 0.012✝
		***Non-Hispanic White***	1.674 ± 0.022✝	1.428 ± 0.032✝	1.706 ± 0.021✝	1.835 ± 0.019✝
		***All Other People***	1.25 ± 0.078✝	--	1.184 ± 0.072✝	1.363 ± 0.073✝
	**Odds of Nutrient Intake Inadequacy given HH Income below $20,000**	**Total Population**	1.034 ± 0.029✝	1.149 ± 0.017✝	1.012 ± 0.03✝	
		***Mexican American***	1.005 ± 0.066✝	0.897 ± 0.045✝	0.965 ± 0.069✝	
		***Other Hispanic***	1.171 ± 0.094✝	1.059 ± 0.058✝	1.09 ± 0.096✝	
		***Non-Hispanic Black***	0.965 ± 0.071✝	1.156 ± 0.05✝	0.983 ± 0.073✝	
		***Non-Hispanic White***	1.047 ± 0.042✝	1.296 ± 0.022✝	1.021 ± 0.043✝	
		***All Other People***	0.958 ± 0.115✝	0.821 ± 0.046✝	0.966 ± 0.119✝	
**Male Cohort**	**RR of Osteoporosis given HH Income below $20,000**	**Total Population**	1.268 ± 0.004✝	1.7 ± 0.003✝	1.393 ± 0.002✝	1.534 ± 0.003✝
		***Mexican American***	1.163 ± 0.003	--	0.881 ± 0.006✝	1.15 ± 0.001✝
		***Other Hispanic***	--	--	--	0.981 ± 0.003
		***Non-Hispanic Black***	2.08 ± 0.012✝	3.121 ± 0.024	2.275 ± 0.018✝	2.057 ± 0.012✝
		***Non-Hispanic White***	1.38 ± 0.013✝	1.464 ± 0.006✝	1.539 ± 0.01✝	1.768 ± 0.008✝
		***All Other People***	2.067 ± 0.065✝	4.385 ± 0.09✝	3.221 ± 0.063✝	1.845 ± 0.04✝
	**Odds of Nutrient Intake Inadequacy given HH Income below $20,000**	**Total Population**	1.195 ± 0.026✝	1.252 ± 0.022✝	1.108 ± 0.029✝	
		***Mexican American***	1.187 ± 0.063✝	1.34 ± 0.059✝	1.119 ± 0.07✝	
		***Other Hispanic***	1.044 ± 0.085✝	0.974 ± 0.07✝	1.036 ± 0.094✝	
		***Non-Hispanic Black***	1.318 ± 0.063✝	1.318 ± 0.056✝	1.205 ± 0.068✝	
		***Non-Hispanic White***	1.182 ± 0.037✝	1.186 ± 0.03✝	1.083 ± 0.042✝	
		***All Other People***	0.893 ± 0.093✝	1.262 ± 0.08✝	0.859 ± 0.104✝	
**All**	**RR of Osteoporosis given HH Income below $20,000**	**Total Population**	1.45 ± 0.008✝	1.414 ± 0.008✝	1.49 ± 0.007✝	1.618 ± 0.007✝
		***Mexican American***	1.695 ± 0.024✝	1.817 ± 0.029✝	1.633 ± 0.02✝	1.681 ± 0.018✝
		***Other Hispanic***	1.16 ± 0.029	0.964 ± 0.023✝	1.222 ± 0.025	1.364 ± 0.024✝
		***Non-Hispanic Black***	1.253 ± 0.006✝	1.128 ± 0.001✝	1.174 ± 0.004✝	1.123 ± 0.003✝
		***Non-Hispanic White***	1.644 ± 0.014✝	1.595 ± 0.016✝	1.736 ± 0.013✝	1.943 ± 0.011✝
		***All Other People***	1.403 ± 0.05✝	1.159 ± 0.05✝	1.52 ± 0.047✝	1.475 ± 0.041✝
	**Odds of Nutrient Intake Inadequacy given HH Income below $20,000**	**Total Population**	1.116 ± 0.02✝	1.189 ± 0.013✝	1.064 ± 0.021✝	
		***Mexican American***	1.105 ± 0.047✝	1.08 ± 0.034✝	1.048 ± 0.05✝	
		***Other Hispanic***	1.132 ± 0.065✝	1.015 ± 0.044✝	1.074 ± 0.068✝	
		***Non-Hispanic Black***	1.12 ± 0.048✝	1.235 ± 0.037✝	1.088 ± 0.05✝	
		***Non-Hispanic White***	1.123 ± 0.029✝	1.22 ± 0.018✝	1.061 ± 0.031✝	
		***All Other People***	0.934 ± 0.076✝	1.047 ± 0.042✝	0.921 ± 0.08✝	

Reported means and standard deviations

✝: indicates statistically significant result (one-tailed p value < 0.25%). “—”indicates insufficient sample sizes for statistical analysis or non-applicable.

#### Annual household income and inadequate calcium intake

Among Americans age of 50 and older, the relative risk of having inadequate calcium intake is 11.6% (RR = 1.116 ± 0.02) greater between those living in households earning less than $20,000 per year versus other households. By gender, risk of inadequate calcium intake in males is 19.5% (RR = 1.195 ± 0.026) greater compared to females whose risk is only 3.4% (RR = 1.034 ± 0.029) greater given annual household income. Considering this low relative risk, females are generally more likely to have inadequate calcium intake independent of income bracket. An exception to this is Other Hispanic females with a 17.1% (RR = 1.171 ± 0.094) greater risk of having inadequate calcium intake given annual household income.

Across ethnicities age 50 and older, low income has the greatest effect on Non-Hispanic Blacks with three-quarters (81.5% of females; 75.6% of males) having inadequate calcium intake (See Table 1). Non-Hispanic black males have the highest relative risk of inadequate calcium intake across ethnicities with a 31.8% (RR = 1.318 ± 0.063) greater risk given annual household income. Conversely, as with the general female population, the relative risk for Non-Hispanic Black females is close to one (RR = 0.965 ± 0.071) indicating inadequate intake levels among this population across all income brackets.

#### Annual household income and inadequate vitamin D intake

Among Americans age 50 and older, the odds of having inadequate vitamin D intake is 18.9% (RR = 1.189 ± 0.013) greater amongst those individuals living in households earning less than $20,000 per year versus other households. Annual household income reflects a larger influence on inadequate vitamin D intake in males versus females. Poverty-stricken males have a 25.2% (RR = 1.252 ± 0.022) greater risk while their poverty-stricken female counterparts have a 14.9% (RR = 1.149 ± 0.017) greater risk of inadequate vitamin D intake given annual household income.

Across ethnicities, the impact of household income on vitamin D intake has the greatest effect on Non-Hispanic Blacks age 50 and older, particularly males. The relative risk of inadequate vitamin D intake is 23.5% (RR = 1.235 ± 0.037) greater amongst individuals in this group given household income. For Non-Hispanic Black males, it is 31.8% (RR = 1.318 ± 0.056) greater between those living in households earning less than $20,000 per year and other households. Mexican American males are also greatly affected with a 34% (RR = 1.34 ± 0.059) greater risk of inadequate vitamin D intake given household income.

The relative risk of inadequate vitamin D intake is also high among Non-Hispanic Whites in the lowest income bracket (22.0%; RR = 1.22 ± 0.018 total; 29.6%; RR = 1.296 ± 0.022 for females) when compared to Non-Hispanic Whites in a higher income bracket. Thus, the evidence suggests that the odds of inadequate vitamin D intake is significantly greater among those living in households earning less than $20,000 per year, independent of biological sex and ethnicity.

#### Annual household income and calcium & vitamin D supplementation

The odds of a given individual age 50 and older supplementing with calcium and vitamin D is slightly greater (6.4%; RR = 1.064 ± 0.021) among individuals living in households earning less than $20,000 per year versus other households. By gender, poorer males have a 10.8% (RR = 1.108 ± 0.029) greater likelihood of supplementation compared to females with a relative risk close to 1 (RR = 1.012 ± 0.03) given annual household income. Among males across ethnicities, Non-Hispanic Blacks have the highest likelihood of supplementation (RR = 1.205 ± 0.068) given household income.

#### Annual household income and the relative risk of osteoporosis

Household income is strongly associated with the relative risk of osteoporosis with 60% (RR = 1.618 ± 0.007) greater relative risk of osteoporosis in individuals age 50 and older with a household income of less than $20,000 versus higher income brackets. By gender, the risk of osteoporosis in males is 53.4% (RR = 1.534 ± 0.003) greater and females are at 50% (RR = 1.507 ± 0.013) greater risk given annual household income.

According to ethnicity, the effect of household income on risk of osteoporosis has the greatest impact on Non-Hispanic Whites of both genders (RR = 1.943 ± 0.011; RR = 1.835 ± 0.019 for females; RR = 1.768 ± 0.008 for males). Lower annual household income does not increase the risk of osteoporosis among Non-Hispanic Black women but does for their male counterparts. The odds of a Non-Hispanic Black male age 50 and older of having osteoporosis from a household earning less than $20,000 is two times (2.057 ± 0.012) greater when compared to individuals from higher income households. This significant finding is reasonable when you consider that the overall risk of probable osteoporosis among Non-Hispanic black males is already very low so a small change in relative risk is expected to result in larger observed differences in odds.

When considering the combined effect of household income and nutrient intake, the relative risk of osteoporosis is greater by 45% (RR = 1.45 ± 0.008) if an individual has inadequate calcium intake; by 41.1% (RR = 1.414 ± 0.008) if inadequate vitamin D intake; and by 49% (1.49 ± 0.007) if supplementing with calcium and vitamin D among individuals with household incomes of less than $20,000 per year compared to other income brackets, independent of biological sex. Among males, the relative risk of osteoporosis is greater by 26.8% (RR = 1.268 ± 0.004) if there is inadequate calcium intake; by 70% (RR = 1.7 ± 0.003) if there is inadequate vitamin D intake; and by 39.3% (1.393 ± 0.002) if supplementing with calcium and vitamin D given household income. Among females, the relative risk of osteoporosis is greater by 44.9% (RR = 1.449 ± 0.014) if calcium intake is inadequate; by 26.4% (RR = 1.264 ± 0.018) if vitamin D intake is inadequate; and by 44.2% (1.442 ± 0.013) if supplementing with calcium and vitamin D given household income.

Findings also show most individuals impacted with osteoporosis age 50 and older have inadequate nutrient intake regardless of income level. An exception is among males in which inadequate vitamin D intake plays a role in the relative risk of osteoporosis (RR = 1.252 ± 0.022) in those males earning less than $20,000 annually. Additionally, among Non-Hispanic Black males with vitamin D intake inadequacy the relative risk is much greater *(*RR = 3.121 ± 0.024) for osteoporosis given household income.

### Poverty levels

It is estimated that over one quarter of Americans age of 50 and older (34.15 million) live below the poverty line. In terms of ethnicity, the total Hispanic population is the highest affected with 37.5% living below the poverty line, followed by 33.5% of Non-Hispanic Blacks and 32% of Other Races over age 50.

Among Americans age of 50 and older living below the poverty line, 68.4% (76.6% of females; 58.2% of males) have inadequate calcium intake; 46.6% (44.3% of females; 49.5% of males) have inadequate vitamin D intake; and only 27.5% (23.2% of females; 34.2% males) use calcium and vitamin D supplements. Within this cohort, 12.6% (17.8% of females; 6.8% of males) have osteoporosis. See Table 4 for the full analysis of the influence of poverty status on the relative risk of calcium and vitamin D intake and osteoporosis with analysis by ethnicity and gender.

**Table 4:** Influence of poverty status on RR of nutrient intake & osteoporosis with analysis by ethnicity and gender, age 50 and older.

Test		Population	Calcium Intake Inadequate, Poverty versus Non-poverty	Vitamin D Intake Inadequate, Poverty versus Non-poverty	Calcium & Vitamin D Supplement Users, Poverty versus Non-poverty	RR of Osteoporosis, Poverty versus Non-poverty
**Female Cohort**	**RR of Osteoporosis given Poverty Status**	**Total Population**	1.005 ± 0.012✝	0.893 ± 0.016✝	1.798 ± 0.03✝	1.144 ± 0.011✝
		***Mexican American***	1.351 ± 0.035✝	1.319 ± 0.046✝	1.714 ± 0.071✝	1.407 ± 0.029✝
		***Other Hispanic***	0.69 ± 0.045✝	0.443 ± 0.049✝	2.9 ± 0.135✝	0.86 ± 0.039✝
		***Non-Hispanic Black***	0.828 ± 0.014✝	0.312 ± 0.015✝	--	0.702 ± 0.012✝
		***Non-Hispanic White***	0.955 ± 0.018✝	1.132 ± 0.03✝	1.958 ± 0.045✝	1.239 ± 0.018✝
		***All Other People***	1.204 ± 0.066✝	--	1.438 ± 0.245✝	1.091 ± 0.06✝
	**Odds of Nutrient Intake Inadequacy given Poverty Status**	**Total Population**	1.059 ± 0.028✝	1.339 ± 0.017✝	0.817 ± 0.016✝	
		***Mexican American***	0.937 ± 0.062✝	1.002 ± 0.043✝	1.285 ± 0.037	
		***Other Hispanic***	1.078 ± 0.085✝	1.145 ± 0.055✝	0.699 ± 0.042✝	
		***Non-Hispanic Black***	1.047 ± 0.069✝	1.182 ± 0.048✝	0.782 ± 0.029✝	
		***Non-Hispanic White***	1.092 ± 0.043✝	1.592 ± 0.025✝	0.81 ± 0.027✝	
		***All Other People***	1.046 ± 0.106✝	1.094 ± 0.048✝	0.591 ± 0.054✝	
**Male Cohort**	**RR of Osteoporosis given Poverty Status**	**Total Population**	2.056 ± 0.006✝	2.716 ± 0.006✝	1.094 ± 0.001✝	1.822 ± 0.004✝
		***Mexican American***	1.25 ± 0.005✝	1.353 ± 0.008✝	--	0.874 ± 0.001✝
		***Other Hispanic***	--	--	1.083 ± 0.057✝	0.955 ± 0.005
		***Non-Hispanic Black***	--	--	--	5.461 ± 0.021
		***Non-Hispanic White***	2.474 ± 0.019✝	2.959 ± 0.014✝	2.277 ± 0.01✝	2.515 ± 0.01✝
		***All Other People***	1.852 ± 0.038✝	--	--	1.398 ± 0.028✝
	**Odds of Nutrient Intake Inadequacy given Poverty Status**	**Total Population**	1.166 ± 0.024✝	1.267 ± 0.021✝	0.826 ± 0.021✝	
		***Mexican American***	1.049 ± 0.053✝	0.969 ± 0.046✝	1.012 ± 0.052✝	
		***Other Hispanic***	1.067 ± 0.076✝	1.114 ± 0.064✝	0.882 ± 0.058✝	
		***Non-Hispanic Black***	1.248 ± 0.06✝	1.18 ± 0.051✝	0.681 ± 0.04✝	
		***Non-Hispanic White***	1.165 ± 0.037✝	1.409 ± 0.032✝	0.814 ± 0.035✝	
		***All Other People***	1.057 ± 0.087✝	1.607 ± 0.07✝	0.947 ± 0.076✝	
**All**	**RR of Osteoporosis given Poverty Status**	**Total Population**	1.16 ± 0.007✝	1.207 ± 0.008✝	1.643 ± 0.011✝	1.305 ± 0.006✝
		***Mexican American***	1.401 ± 0.02	1.456 ± 0.024✝	1.575 ± 0.023✝	1.434 ± 0.014✝
		***Other Hispanic***	0.691 ± 0.024✝	0.41 ± 0.018✝	1.735 ± 0.053✝	0.859 ± 0.019✝
		***Non-Hispanic Black***	1.268 ± 0.007✝	0.929 ± 0.001✝	--	1.135 ± 0.004✝
		***Non-Hispanic White***	1.247 ± 0.013✝	1.548 ± 0.015✝	2.108 ± 0.019✝	1.534 ± 0.01✝
		***All Other People***	1.258 ± 0.04✝	1.1 ± 0.04✝	0.857 ± 0.073✝	1.117 ± 0.032✝
	**Odds of Nutrient Intake Inadequacy given Poverty Status**	**Total Population**	1.11 ± 0.019✝	1.298 ± 0.013✝	0.811 ± 0.013✝	
		***Mexican American***	1.002 ± 0.041✝	0.991 ± 0.031✝	1.066 ± 0.031✝	
		***Other Hispanic***	1.069 ± 0.058✝	1.131 ± 0.041✝	0.812 ± 0.035✝	
		***Non-Hispanic Black***	1.144 ± 0.046✝	1.179 ± 0.034✝	0.694 ± 0.025	
		***Non-Hispanic White***	1.131 ± 0.029✝	1.484 ± 0.019✝	0.803 ± 0.022✝	
		***All Other People***	1.038 ± 0.069✝	1.37 ± 0.041✝	0.821 ± 0.046✝	

Poverty Status is measured by Monthly Poverty Level Index < = 1.3. Reported sample means and standard deviations

✝: indicates statistically significant result (one-tailed p value < 0.25%). “—”indicates insufficient sample sizes for statistical analysis or non-applicable.

#### Poverty and inadequate calcium intake

**Fig 1:**
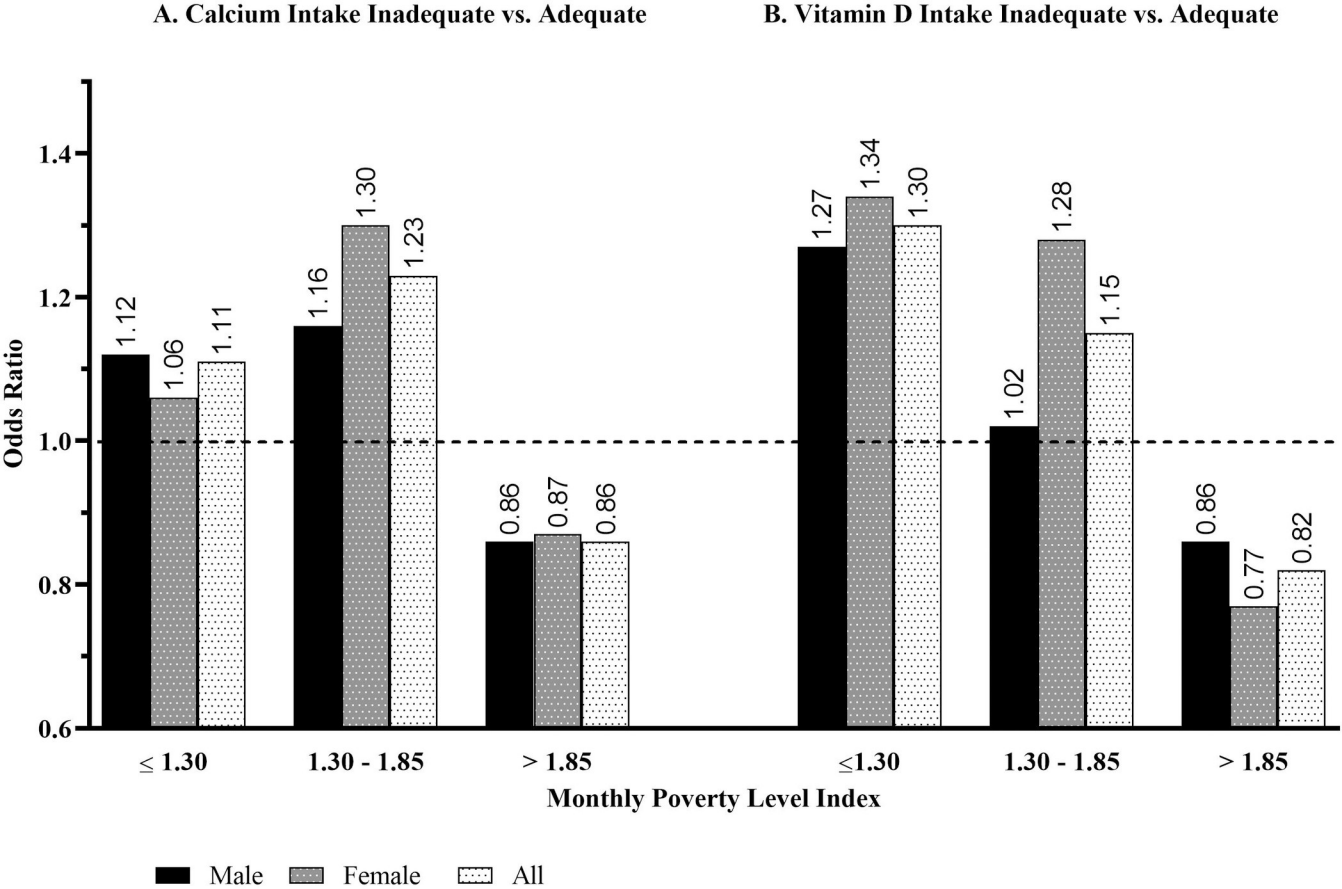
Odds of nutrient intake inadequacy given monthly poverty level index category levels, age 50 and older.

The odds of an American individual age 50 and older having inadequate calcium intake is 11.0% greater (RR = 1.11 ± 0.019) among those that live below the poverty line versus above it. By gender, risk of inadequate calcium intake in males is 16.6% (RR = 1.166 ± 0.024) greater compared to females with a 5.9% (RR = 1.059 ± 0.028) greater risk given poverty status. This again indicates that most females, independent of poverty status, are likely calcium intake inadequate. As shown in Fig 1A, the odds of an American individual age 50 and older having inadequate calcium intake remains significantly greater than those who are in the near-poverty category, which is defined as an individual that had a reported monthly poverty level index value of greater than 1.3 but less than and equal to 1.8. Furthermore, the odds of an American individual age 50 or older that has inadequate calcium intake is significantly less than among those who had a reported monthly poverty level index greater than 1.8. Thus, the odds that an American has inadequate calcium intake is much lower for those who do not live in a state of abject, or near, poverty compared to those who do.

Non-Hispanic Blacks within this cohort are most affected with 80.9% having inadequate calcium intake (85.9% of females; 73.6% of males). They have 14.4% (RR = 1.144 ± 0.046) greater odds of inadequate calcium intake given their poverty status. Non-Hispanic Black males are particularly affected with 24.8% (RR = 1.248 ± 0.06) at greater risk of having inadequate calcium intake.

Non-Hispanic Whites are also impacted by living below the poverty line with a 13.1% (RR = 1.131 ± 0.029) greater relative risk of inadequate calcium intake among all Americans age 50 or older; 16.5% (RR = 1.165 ± 0.037) greater risk among males; and 9.2% (RR = 1.092 ± 0.043) greater risk among females of those living below the poverty line versus above it.

#### Poverty and inadequate vitamin D intake

The odds of an American individual age 50 and older having inadequate vitamin D intake is almost 30% (RR = 1.298 ± 0.013) greater among those that live below the poverty line versus above it. By gender, risk of inadequate vitamin D intake in males is 26.7% (RR = 1.267 ± 0.021) greater compared to females with only a 33.9% (RR = 1.339 ± 0.017) greater risk in those females living below the poverty line versus those living above. Furthermore, the odds of an American individual age 50 and older having inadequate vitamin D intake remains significantly greater than one among those who are in the near-poverty category as shown in Fig 1B. Also, the odds of an American individual age 50 and older having inadequate vitamin D intake is significantly less than among those who had a reported monthly poverty level index value of greater than 1.8. Thus, the odds that an American has inadequate vitamin D intake is much lower in occurrence among those individuals who do not live in a state of poverty.

In terms of ethnicity, Non-Hispanic White individuals living below the poverty line have 48.4% (RR = 1.484 ± 0.019) greater odds (RR = 1.409 ± 0.032 for males; RR = 1.592 ± 0.025 for females) of having inadequate vitamin D intake compared to those living above the poverty line.

As demonstrated with calcium intake inadequacy, the majority of Non-Hispanic Blacks living below the poverty line are impacted by vitamin D intake inadequacy (57.2% total; 56.3% of females; 58.5% of males). However, in terms of the impact of poverty on the relative risk of inadequate vitamin D intake, the relative risk is lower compared to the population as a whole (RR = 1.179 ± 0.034 for Non-Hispanic Blacks versus RR = 1.298 ± 0.013 for total population). This could mean that Non-Hispanic Blacks generally have inadequate vitamin D intake regardless of poverty status.

#### Poverty and calcium & vitamin D supplementation

The odds of an American age 50 and older supplementing with calcium and vitamin D is 19% (RR = 0.811 ± 0.013) less among those that live below versus above the poverty line. This trend is generally consistent across both genders and all ethnicities. Poverty has the greatest impact on females of Other Races who are 40.9% (RR = 0.591 ± 0.054) less likely to supplement with vitamin D and calcium if living below the poverty line.

#### Poverty and the relative risk of osteoporosis

Like household income, poverty status is also strongly associated with the relative risk of osteoporosis. An individual age of 50 and older living below the poverty line has a 30.5% (RR = 1.305 ± 0.006) greater relative risk of osteoporosis in comparison to individuals living above the poverty line. By gender, the risk of osteoporosis in males is 82.2% (RR = 1.822 ± 0.004) greater compared to females with only a 14.4% (RR = 1.144 ± 0.011) greater risk given poverty status.

When analyzing the data according to ethnicity, the effect of poverty status on risk of osteoporosis has the greatest impact on Mexican Americans, specifically females (RR = 1.434 ± 0.014; RR = 1.407 ± 0.029 for females); and Non-Hispanic Whites of both genders (RR = 1.534 ± 0.01; RR = 1.239 ± 0.018 for females; RR = 2.515 ± 0.01 for males).

In contrast, the relative risk of osteoporosis is 29.8% (RR = 0.702 ± 0.012) less among Non-Hispanic Black females age 50 and older who live below the poverty line. While the prevalence of osteoporosis amongst females of Other Races living below the poverty line is almost 30%, the relative risk is only 9.1% (RR = 1.091 ± 0.06) between poverty-stricken females of Other Races versus their non-poverty counterparts. This suggests that osteoporosis has a similar prevalence among women of Other Races regardless of poverty status.

**Fig 2:**
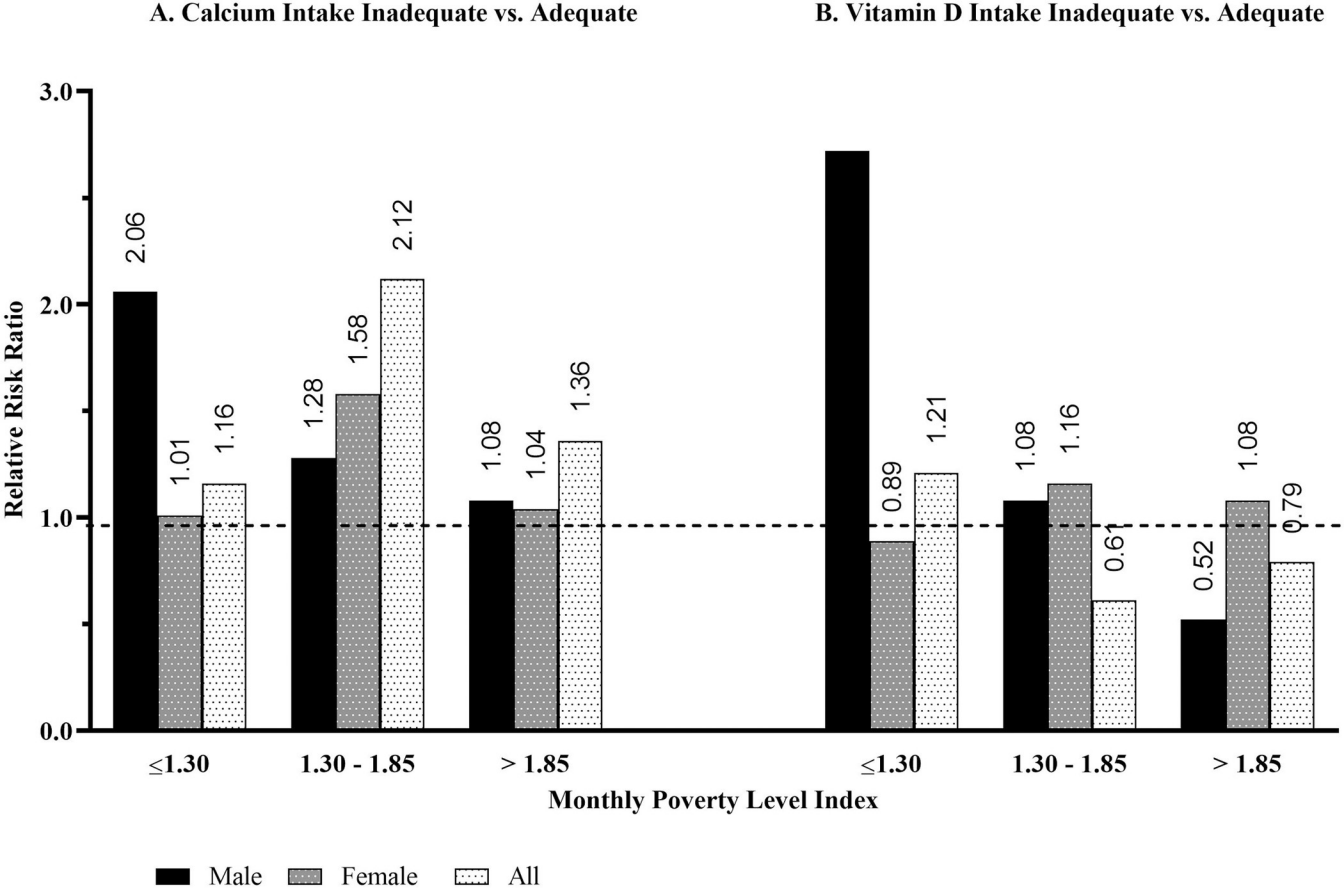
Relative risk of osteoporosis given monthly poverty level index category levels, age 50 and older.

When considering the combined effect of poverty and nutrient intake on the relative risk of osteoporosis, a given individual living below the poverty line versus their non-poverty counterparts has a 16% greater relative risk (RR = 1.16 ± 0.007) if they have inadequate calcium intake; 20.7% (RR = 1.207 ± 0.008) greater risk if they have inadequate vitamin D intake; and a 64.3% (RR = 1.643 ± 0.011) higher likelihood if supplementing with calcium and vitamin D. Among males, the relative risk of osteoporosis is two times (RR = 2.056 ± 0.006) greater if they have inadequate calcium intake and 2.7 times (RR = 2.716 ± 0.006) greater if have inadequate vitamin D intake. Among females, the relative risk of osteoporosis is less than 1% (RR = 1.005 ± 0.012) greater if calcium intake is inadequate but 9.1% (RR = 0.893 ± 0.016) less if vitamin D intake is inadequate. These mixed results are likely due to the high levels of nutrient intake inadequacies being high among females independent of poverty level. As shown in Fig 2A, the relative risk of osteoporosis among Americans age of 50 and older with inadequate calcium or vitamin D intake remains greater than among those who are in the near-poverty category of greater than 1.3 but less than and equal to 1.8. Furthermore, the relative risk of osteoporosis of an American individual age 50 and older having inadequate calcium intake remains greater than one among those who had a reported monthly poverty level index value of greater than 1.8, but it is smaller in magnitude compared to those who are in the near-poverty category. A clear decline in relative risk of osteoporosis is observed among males with inadequate calcium or vitamin D intake as income level increases.

Females have a 79.8% (RR = 1.798 ± 0.03) higher likelihood of supplementing with calcium and vitamin D if they live below the poverty line. Men have a less than 10% (RR = 1.094 ± 0.001) higher likelihood of supplementing with calcium and vitamin D if they live below the poverty line. These surprising results suggests that the likelihood of a given individual being a user of a calcium and vitamin D supplement is greater among those that live below the poverty line. As shown above, the odds of a given individual having a calcium and vitamin D intake inadequacy does increase with a decrease in poverty level which suggests that this population may be more likely to be currently using supplementation as a means to address nutrient intake gaps.

When considering the combined effect of poverty and nutrient intake on relative risk of osteoporosis, it appears for females, inadequate calcium and/or vitamin D intake does not play a strong role given poverty status. The findings indicate that individuals impacted with osteoporosis age 50 and older have inadequate nutrient intakes regardless of income level. However, the likelihood of supplementation is greater, perhaps indicating that those with osteoporosis are supplementing as a treatment. This trend is consistent with Mexican Americans and Non-Hispanic white females living below the poverty line.

Conversely, when performing the same analysis for males, calcium and vitamin D intake inadequacy plays a role in the relative risk of osteoporosis among those below the poverty line compared to those living above. However, having osteoporosis does not increase supplementation among poverty-stricken males. In the case of Non-Hispanic White males over the age of 50 living in poverty, only inadequate vitamin D intake seems to have an impact on the relative risk of osteoporosis (RR = 2.959 ± 0.014 for RR of osteoporosis with vitamin D intake inadequacy considering poverty status). As well, this cohort diagnosed with osteoporosis seems more likely to supplement (RR = 2.277 ± 0.01 for risk of osteoporosis with supplementation considering poverty status).

### Food insecurity

NHANES provides a metric (FSDHH) that attempts to quantify the level of food insecurity a given household faces called the Household Food Security index. This measurement is based on a self-reported question to individual survey participants on their perceived level of food security in terms of missed meals in a given month. Thus, this measure is more concerned with access to food in general as opposed to access to nutritious food. Though this measurement of food insecurity is highly correlated with household income and poverty levels, there are likely other influential factors that may impact food insecurity including cultural differences in what qualifies as a meal, differences in what qualifies as a nutritious meal and differences in access to nutritious food. Just over 11% of Americans (11.9% females, 10.4% males) over the age of 50 report being food insecure. In terms of ethnicity, food insecurity impacts 23.6% of Total Hispanics and 17.8% of Non-Hispanic Blacks age 50 and older.

Among Americans age 50 and older reporting food insecurity, 68.4% (76.2% females, 58.4% males) have inadequate calcium intake; half (48.4% females, 52% males) have inadequate vitamin D intake; and only a quarter (20% of females; 15.5% of males) use calcium and vitamin D supplements. Over 11.2% (17.4% of females; 4.2% of males) of this cohort are affected by osteoporosis. See Table 5 for the full analysis of the influence of food security status on the relative risk of calcium and vitamin D intake and osteoporosis with analysis by ethnicity and gender.

**Table 5:** Influence of food security status on RR of nutrient intake & osteoporosis with analysis by ethnicity and gender, age 50 and older.

Test		Population	Calcium Intake Inadequate, Poverty versus Non-poverty	Vitamin D Intake Inadequate, Poverty versus Non-poverty	Calcium & Vitamin D Supplement Users, Poverty versus Non-poverty	RR of Osteoporosis, Poverty versus Non-poverty
**Female Cohort**	**RR of Osteoporosis given Food Insecurity**	**Total Population**	0.961 ± 0.014✝	0.774 ± 0.012✝	0.936 ± 0.013✝	1.086 ± 0.013✝
		***Mexican American***	1.032 ± 0.037✝	0.984 ± 0.044✝	0.963 ± 0.033✝	1.261 ± 0.033✝
		***Other Hispanic***	0.608 ± 0.039✝	0.237 ± 0.035✝	0.562 ± 0.036✝	0.639 ± 0.031✝
		***Non-Hispanic Black***	0.803 ± 0.011✝	--	0.748 ± 0.009✝	0.674 ± 0.007✝
		***Non-Hispanic White***	1.425 ± 0.033✝	1.245 ± 0.037✝	1.48 ± 0.032✝	1.561 ± 0.03✝
		***All Other People***	0.439 ± 0.055✝	--	0.44 ± 0.053✝	0.667 ± 0.062✝
	**Odds of Nutrient Intake Inadequacy given Food Insecurity**	**Total Population**	1.034 ± 0.036✝	1.381 ± 0.024✝	1.046 ± 0.037✝	
		***Mexican American***	0.913 ± 0.072✝	1.012 ± 0.051✝	0.952 ± 0.076✝	
		***Other Hispanic***	1.054 ± 0.094✝	1.462 ± 0.066✝	1.141 ± 0.099✝	
		***Non-Hispanic Black***	0.999 ± 0.083✝	1.023 ± 0.055✝	0.979 ± 0.084✝	
		***Non-Hispanic White***	1.068 ± 0.064✝	1.754 ± 0.043✝	1.048 ± 0.066✝	
		***All Other People***	1.052 ± 0.143✝	0.973 ± 0.06✝	1.01 ± 0.144✝	
**Male Cohort**	**RR of Osteoporosis given Food Insecurity**	**Total Population**	0.669 ± 0.007✝	0.802 ± 0.014✝	0.864 ± 0.003✝	0.898 ± 0.006✝
		***Mexican American***	--	--	--	--
		***Other Hispanic***	--	--	--	0.925 ± 0.019
		***Non-Hispanic Black***	--	--	--	--
		***Non-Hispanic White***	1.599 ± 0.027✝	1.139 ± 0.013✝	1.826 ± 0.02✝	1.788 ± 0.015✝
		***All Other People***	--	5 ± 0.183✝	3.2 ± 0.103✝	1.613 ± 0.062✝
	**Odds of Nutrient Intake Inadequacy given Food Insecurity**	**Total Population**	1.16 ± 0.032✝	1.289 ± 0.029✝	1.105 ± 0.036✝	
		***Mexican American***	1.293 ± 0.065✝	1.269 ± 0.057✝	1.16 ± 0.07✝	
		***Other Hispanic***	0.986 ± 0.084✝	1.095 ± 0.074✝	0.938 ± 0.092✝	
		***Non-Hispanic Black***	1.101 ± 0.076✝	1.098 ± 0.067✝	1.09 ± 0.083✝	
		***Non-Hispanic White***	1.092 ± 0.057✝	1.431 ± 0.055✝	1.115 ± 0.067✝	
		***All Other People***	1.068 ± 0.14✝	1.076 ± 0.108✝	1.008 ± 0.152✝	
**All**	**RR of Osteoporosis given Food Insecurity**	**Total Population**	0.9 ± 0.007✝	0.817 ± 0.003✝	0.931 ± 0.006✝	1.074 ± 0.006✝
		***Mexican American***	0.702 ± 0.016✝	0.659 ± 0.012✝	0.671 ± 0.012✝	0.94 ± 0.013✝
		***Other Hispanic***	0.584 ± 0.021✝	0.262 ± 0.029✝	0.57 ± 0.015✝	0.69 ± 0.017✝
		***Non-Hispanic Black***	0.856 ± 0.003✝	0.593 ± 0.032✝	0.81 ± 0.002✝	0.758 ± 0.002✝
		***Non-Hispanic White***	1.499 ± 0.023✝	1.361 ± 0.016✝	1.593 ± 0.02✝	1.681 ± 0.017✝
		***All Other People***	0.614 ± 0.039✝	1.367 ± 0.066✝	0.838 ± 0.041✝	0.895 ± 0.041✝
	**Odds of Nutrient Intake Inadequacy given Food Insecurity**	**Total Population**	0.184 ± 0.013✝	0.225 ± 0.014✝	0.182 ± 0.012✝	
		***Mexican American***	0.324 ± 0.031✝	0.347 ± 0.032✝	0.317 ± 0.03✝	
		***Other Hispanic***	0.324 ± 0.037	0.401 ± 0.04✝	0.332 ± 0.036✝	
		***Non-Hispanic Black***	0.225 ± 0.028	0.225 ± 0.03	0.221 ± 0.027	
		***Non-Hispanic White***	0.099 ± 0.019	0.142 ± 0.023	0.098 ± 0.018	
		***All Other People***	0.158 ± 0.046✝	0.146 ± 0.06✝	0.149 ± 0.044✝	

Reported means and standard deviations

✝: indicates statistically significant result (one-tailed p value < 0.25%). “—”indicates insufficient sample sizes for statistical analysis or non-applicable.

#### Food insecurity and inadequate calcium intake

The odds of an individual age 50 and older having inadequate calcium intake is significantly less (RR = 0.184 ± 0.013) between food insecure and food secure individuals, independent of ethnicity and biological sex. However, risk of inadequate calcium intake in males is 16% (RR = 1.16 ± 0.032) greater compared to females with only a 3.4% (RR = 1.034 ± 0.036) greater risk given annual household income. These mixed results suggest that food insecurity, and its consequential effect, may impact males and females differently. While over three-quarters of food insecure females age 50 and older are impacted by inadequate calcium intake, the relative risk is generally close to one across ethnicities, suggesting calcium intake inadequacy occurs among females regardless of food security status or ethnic group. Among males, Mexican Americans are most affected with a 29.3% (RR = 1.293 ± 0.065) greater risk of having calcium inadequate intake if they are food insecure compared to their food secure counterparts.

#### Food insecurity and vitamin D intake inadequacy

Like the case of inadequate calcium intake, the odds of an individual age 50 and having inadequate vitamin D intake is less between those reporting food insecurity and those reporting being food secure (RR = 0.225 ± 0.014). By gender, risk of inadequate calcium intake in males is 28.9% (RR = 1.289 ± 0.029) greater and in females 38.1% (RR = 1.381 ± 0.024) greater between individuals who are food insecure and food secure. Thus, these results again suggest that the consequential effects of may impact males and females differently.

#### Food insecurity and calcium & vitamin D supplementation

The odds of an American age 50 and older supplementing with calcium and vitamin D is significantly less likely (RR = 0.182 ± 0.012) among individuals who report food insecurity versus food security. However, the likelihood of supplementation in males is 10.5% (RR = 1.105 ± 0.036) greater in food insecure compared to food secure individuals. In contrast, food security status does not play a role in supplementation among females as the relative risk of supplementation is just over 1.

*Food insecurity and the relative risk of osteoporosis*. Food insecurity status does not seem to play a strong role in the relative risk of osteoporosis. An individual age 50 and older reporting food insecurity has a 7.4% (RR = 1.074 ± 0.006) greater relative risk of osteoporosis than an individual reporting food security. By gender, the risk of osteoporosis in males is 10% (RR = 0.898 ± 0.006) less compared to females with 8.6% (RR = 1.086 ± 0.013) greater risk given food insecurity status.

When considering ethnicity, Non-Hispanic White food insecurity influences the relative risk of osteoporosis with those reporting food insecurity having a 68.1% (RR = 1.681 ± 0.017; 1.561 ± 0.03 for females; 1.788 ± 0.015 for males) greater relative risk of osteoporosis compared to their food secure counterparts. Mexican American female food insecure individuals are also impacted with a 26% (1.261 ± 0.033) greater risk of osteoporosis given their food insecurity.

Considering the combined effect of food insecurity and nutrient intake, the relative risk of osteoporosis in a food insecure individual versus their food secure counterparts is 10% (RR = 0.9 ± 0.007) less if they have inadequate calcium intake; 18.3% (RR = 0.817 ± 0.003) less if they have inadequate vitamin D intake; and there is a 6.9% (RR = 0.931 ± 0.006) less likelihood of supplementing with calcium and vitamin D. Among females, the relative risk of osteoporosis is 3.9% (RR = 0.961 ± 0.014) less if inadequate calcium intake; 22.6% (RR = 0.774 ± 0.012) less if inadequate vitamin D; and there is a 6.4% (0.936 ± 0.013) less likelihood if supplementing with calcium and vitamin D given food security status. Among males, the relative risk of osteoporosis is 33.1% (RR = 0.669 ± 0.007) less if inadequate calcium intake; 19.8% (RR = 0.802 ± 0.014) less if inadequate vitamin D intake; and there is a 13.6% (0.864 ± 0.003) less likelihood if supplementing with calcium and vitamin D. When considering the combined effect of food security and nutrient intake on the relative risk of osteoporosis, it appears inadequate calcium and vitamin D intake does increase the relative risk given food insecurity status. Osteoporosis does not appear to affect supplementation rates. One exception is Non-Hispanic White males with osteoporosis are more likely to supplement with calcium and vitamin D.

### SNAP participation

SNAP participation, while it is an indicator of poverty, it is also an indication that the participating individual likely has improved access to food and nutrition and greater opportunities for participants to fill any nutritional intake gaps. This in turn will likely lead to more varied results when compared to other measurements of poverty. An estimated 12% of Americans (just under 16 million) age 50 and older participate in the SNAP program (12.3% of females, 11.5% of males). In terms of ethnicity, 22.1% of Non-Hispanic Blacks and 17.1% Total Hispanics age 50 and older are SNAP participants.

Among Americans age 50 and older participating in the SNAP program, 65.6% (77.3% of females; 51.3% of males) have inadequate calcium intake; 46.0% (47.8% of females, 43.8% of males) have inadequate vitamin D intake; and 28.6% (19.1% of females, 40.2% of males) use calcium and vitamin D supplements. Almost 12% (15.3% of females; 7.9% of males) are impacted by osteoporosis. See Table 6 for the full analysis of the influence of SNAP participation on the relative risk of calcium and vitamin D intake and osteoporosis with analysis by ethnicity and gender.

**Table 6:** Influence of SNAP participation on RR of nutrient intake & osteoporosis with analysis by ethnicity and gender, age 50 and older.

Test		Population	Calcium Intake Inadequate, Poverty versus Non-poverty	Vitamin D Intake Inadequate, Poverty versus Non-poverty	Calcium & Vitamin D Supplement Users, Poverty versus Non-poverty	RR of Osteoporosis, Poverty versus Non-poverty
**Female Cohort**	**RR of Osteoporosis given SNAP participation**	**Total Population**	0.992 ± 0.013	0.998 ± 0.015	0.522 ± 0.012✝	0.937 ± 0.011✝
		***Mexican American***	1.723 ± 0.053✝	1.538 ± 0.058✝	--	1.132 ± 0.033✝
		***Other Hispanic***	0.239 ± 0.025✝	--	--	0.313 ± 0.021✝
		***Non-Hispanic Black***	1.229 ± 0.012✝	--	--	1.103 ± 0.009✝
		***Non-Hispanic White***	1.339 ± 0.027✝	1.435 ± 0.037✝	0.669 ± 0.023✝	1.365 ± 0.023✝
		***All Other People***	--	--	--	0.5 ± 0.063✝
	**Odds of Nutrient Intake Inadequacy given SNAP participation**	**Total Population**	1.052 ± 0.036✝	1.33 ± 0.022✝	0.849 ± 0.015✝	
		***Mexican American***	0.884 ± 0.075✝	1.111 ± 0.055✝	1.399 ± 0.042✝	
		***Other Hispanic***	0.984 ± 0.102✝	0.907 ± 0.057✝	1.184 ± 0.043✝	
		***Non-Hispanic Black***	1.098 ± 0.08✝	1.138 ± 0.052✝	0.607 ± 0.015✝	
		***Non-Hispanic White***	1.083 ± 0.06✝	1.707 ± 0.038✝	0.761 ± 0.027✝	
		***All Other People***	1.015 ± 0.169✝	0.463 ± 0.022✝	1.096 ± 0.082✝	
**Male Cohort**	**RR of Osteoporosis given SNAP participation**	**Total Population**	1.558 ± 0.003✝	2.6 ± 0.007✝	2.024 ± 0.006✝	1.965 ± 0.003✝
		***Mexican American***	--	--	--	--
		***Other Hispanic***	--	--	5.6 ± 0.082✝	5.164 ± 0.029✝
		***Non-Hispanic Black***	6.556 ± 0.036✝	--	--	4.793 ± 0.026
		***Non-Hispanic White***	1.599 ± 0.02✝	1.955 ± 0.009✝	3.105 ± 0.013✝	2.1 ± 0.01✝
		***All Other People***	2.25 ± 0.057✝	6.15 ± 0.113✝	--	1.41 ± 0.027✝
	**Odds of Nutrient Intake Inadequacy given SNAP participation**	**Total Population**	1.014 ± 0.03✝	1.089 ± 0.027✝	1.068 ± 0.023✝	
		***Mexican American***	0.81 ± 0.066✝	1.093 ± 0.072✝	1.194 ± 0.065✝	
		***Other Hispanic***	0.98 ± 0.088✝	0.837 ± 0.074✝	1.476 ± 0.065✝	
		***Non-Hispanic Black***	1.097 ± 0.066✝	1.013 ± 0.058✝	0.944 ± 0.035✝	
		***Non-Hispanic White***	0.915 ± 0.047✝	1.122 ± 0.045✝	1.099 ± 0.046✝	
		***All Other People***	0.94 ± 0.1✝	1.032 ± 0.083✝	1.33 ± 0.084✝	
**All**	**RR of Osteoporosis given SNAP participation**	**Total Population**	1.104 ± 0.007✝	1.347 ± 0.007✝	0.857 ± 0.002✝	1.141 ± 0.005✝
		***Mexican American***	1.695 ± 0.031✝	1.621 ± 0.03✝	--	1.181 ± 0.015✝
		***Other Hispanic***	0.327 ± 0.013✝	--	2.267 ± 0.053✝	0.727 ± 0.014✝
		***Non-Hispanic Black***	1.766 ± 0.006✝	1.373 ± 0.01✝	--	1.554 ± 0.003✝
		***Non-Hispanic White***	1.455 ± 0.018	1.775 ± 0.02✝	1.157 ± 0.008✝	1.576 ± 0.013✝
		***All Other People***	0.643 ± 0.033✝	2.278 ± 0.086✝	--	0.646 ± 0.026✝
	**Odds of Nutrient Intake Inadequacy given SNAP participation**	**Total Population**	0.172 ± 0.012✝	0.199 ± 0.014✝	0.975 ± 0.014✝	
		***Mexican American***	0.169 ± 0.031	0.212 ± 0.031	1.215 ± 0.037✝	
		***Other Hispanic***	0.224 ± 0.036	0.2 ± 0.04	1.375 ± 0.039✝	
		***Non-Hispanic Black***	0.311 ± 0.027✝	0.304 ± 0.028✝	0.824 ± 0.02✝	
		***Non-Hispanic White***	0.109 ± 0.019	0.146 ± 0.023	0.934 ± 0.026✝	
		***All Other People***	0.151 ± 0.045✝	0.146 ± 0.058✝	1.394 ± 0.061✝	

Reported means and standard deviations

✝: indicates statistically significant result (one-tailed p value < 0.25%). “—”indicates insufficient sample sizes for statistical analysis or non-applicable.

#### SNAP participation and inadequate calcium intake

Participation in the SNAP program does not seem to increase the odds of inadequate calcium intake (RR = 0.172 ± 0.012). This trend is largely consistent across ethnicities and both genders and suggests that better access to food and nutrition and allows for greater opportunities to fill any nutritional intake gaps. Non-Hispanic Black female and male SNAP participants are exceptions. Almost 90% of female and 68% of male Non-Hispanic Black SNAP participants have inadequate calcium intake. Both genders have an approximate 10% (RR = 1.098 ± 0.08 for females; RR = 1.097 ± 0.066 for males) greater relative risk of having inadequate calcium intake compared to their non-SNAP counterparts. SNAP participation also impacts Non-Hispanic White females who have an 8.3% (RR = 1.083 ± 0.06) greater relative risk of inadequate calcium intake if participating in SNAP versus their non-SNAP counterparts.

#### SNAP participation and inadequate vitamin D intake

Participation in the SNAP program does not seem to increase relative risk of inadequate vitamin D intake with a relative risk of 0.199 (± 0.014) among all SNAP participants and 1.089 (± 0.027) among male SNAP participants when compared to non-SNAP participants.

SNAP participation has a greater influence on vitamin D intake status among females, particularly Non-Hispanic white females. The odds of a female age 50 and older with inadequate vitamin D intake is 33% (RR = 1.33 ± 0.022) greater (70.7% greater for Non-Hispanic White females; RR = 1.707 ± 0.038) given SNAP participation.

#### SNAP participation and calcium and vitamin D supplementation

When considering the entire cohort, SNAP participation status again does not influence vitamin D and calcium supplement use (RR = 0.975 ± 0.014). This is consistent when analyzing genders separately. In terms of ethnicity, sample sizes were too small to give reliable results but overall, the findings are consistent with expectations that individuals with lower income levels and that rely on government assistance for food and nutrition are likely to use a dietary supplement product.

#### SNAP participation and the relative risk of osteoporosis

The odds for an individual age 50 and older being at risk for osteoporosis is 14.1% (RR = 1.141 ± 0.005) greater than individuals participating in the SNAP program versus non-SNAP participants. SNAP participation strongly influences the risk of osteoporosis in males and yet has no effect for females. The risk of osteoporosis in males is 96% (1.965 ± 0.003) greater compared to females with only 1% (0.937 ± 0.011) less risk given annual household income.

When analyzing the data according to ethnicity, however, the effect of SNAP participation on risk of osteoporosis has the greatest impact on Non-Hispanic Whites (RR = 1.576 ± 0.013; RR = 1.365 ± 0.023 for females; RR = 2.1 ± 0.01 for males). Mexican American females are also impacted by osteoporosis, with 13.2% (RR = 1.132 ± 0.033) greater relative risk among SNAP participants compared to their non-SNAP counterparts. Among males, SNAP participation is correlated with a higher relative risk of osteoporosis in males across all ethnicities. Other Hispanic male SNAP participants are five times (RR = 5.164 ± 0.029) more likely to have osteoporosis versus their non-SNAP counterparts.

Within the Non-Hispanic Black community, only 7.1% of SNAP participants have osteoporosis. However, in terms of relative risk, Non-Hispanic Black SNAP participants have a 55.4% (RR = 1.554 ± 0.003) greater relative risk of osteoporosis versus their non-SNAP counterparts. In contrast, despite inadequate calcium and vitamin D intake as well as decreased likelihood of supplement use, only 9.1% of Non-Hispanic Black female SNAP participants have osteoporosis, and they have a 10% (1.103 ± 0.009) greater relative risk of osteoporosis versus their non-SNAP counterparts.

The combined effect of SNAP participation and nutrient intake on the relative risk of osteoporosis in a SNAP participant versus non-SNAP counterpart is 10.4% (RR = 1.104 ± 0.007) greater if they have inadequate calcium intake; 34.7%% (RR = 1.347 ± 0.007) greater if they have inadequate vitamin D; and there is a 14.3% (RR = 0.857 ± 0.002) less likelihood if supplementing with calcium and vitamin D. Among females, the relative risk of osteoporosis with calcium or vitamin D intake given SNAP participation was not statistically significant and there is a 44.8% (RR = 0.522 ± 0.012) less likelihood of osteoporosis if supplementing with calcium and vitamin D given SNAP participation status. Among males, the relative risk of osteoporosis is 55.8% (RR = 1.558 ± 0.003) greater if inadequate calcium intake; 2.5 times (RR = 2.6 ± 0.007) greater if inadequate vitamin D intake; and there is two times (RR = 2.024 ± 0.006) greater likelihood of osteoporosis if supplementing with calcium and vitamin D given SNAP participation status. When considering the combined effect of SNAP participation and nutrient intake on the relative risk of osteoporosis, it appears that inadequate vitamin D intake plays a role given SNAP participation. This trend is consistent across both genders and among Non-Hispanic Whites.

Exceptions to these results are among Non-Hispanic Blacks, with a 76.6% (RR = 1.766 ± 0.006; RR = 1.229 ± 0.012 for females) increased relative risk of osteoporosis in individuals with inadequate calcium intake between SNAP participants versus non-SNAP participants. Inadequate calcium intake also increases the risk of osteoporosis among Mexican American female SNAP participants (RR = 1.723 ± 0.053) compared to their non-SNAP counterparts. Osteoporosis among all male SNAP participants as well as Non-Hispanic white males is associated with increased supplementation.

## Discussion & conclusion

This investigation builds upon earlier work on bone health nutrient disparities in US subpopulations [7] with inclusion of the latest US population data (NHANES data) and detailed evaluation of economic disparities in relation to inadequate nutrient intake and osteoporosis risk. In this analysis, we found poverty—in particular household income and poverty status—to be a strong indicator for risk of osteoporosis. All four poverty indicators used in this analysis were strong indicators for osteoporosis in Non-Hispanic Whites, as well as Mexican American females. While in general, the prevalence for osteoporosis is four times greater among females than males age 50 and older regardless of poverty status, poverty strongly increases the risk of osteoporosis among males.

Individuals with low income, poverty and food insecurity showed an increased incidence of inadequate calcium intake. Our findings are similar to those observed by Wallace et al, which noted low-income populations may be a greater risk of inadequate calcium and vitamin D intakes [7]. While inadequate calcium intake has a larger prevalence among females than males, poverty markers among males increased the risk of inadequate calcium intake more than females, with the exception of the SNAP program. This is potentially due to the fact that females have inadequate calcium intake regardless of poverty status. SNAP participation status increases the risk for both males and females when analyzing separately by gender.

The combined effect of poverty markers and nutrient intake on the relative risk of osteoporosis was mixed: inadequate vitamin D intake is consistently connected to the risk of osteoporosis among poverty-stricken individuals, although inadequate calcium intake does play a role in certain cases. Recent work, by Cowan et al., also showed varied findings for relationship between inadequate nutrient intake and food security, noting higher prevalence of inadequacy for food insecure individuals for some nutrients such as vitamins C, D, E, and K but not for others such as calcium and folate [14].

In general, this analysis suggests that nutrient deficiencies affect poverty-stricken males more than females in relation to the risk of osteoporosis. This could be due to the fact that other gender specific health issues play a more dominant role in osteoporosis risk for females such as menopause whereas major osteoporosis risk factors for men are age and inadequate nutrition, which can be exacerbated by economic disparities.

When assessing adequacy of nutrient intake, access across all options including diet, fortification and supplementation use should be considered. In the US, calcium and vitamin D fortification has evolved over time and is now readily found in a variety of foods, including milk and breakfast items, such as cereal. Additionally, dietary supplement use in the US has increased over time leading to increased nutrient intakes. Calcium and vitamin D supplementation among low income populations versus financially stable populations is mixed. Generally, poverty indicators are associated with less likelihood of nutrient supplementation, with the exception of males who are low-income or food insecure. SNAP participation status also does not influence the likelihood of supplementation. When considering the combined effect of supplementation and poverty, this analysis found that supplementation use is often stronger among those with osteoporosis, particularly among Non-Hispanic white males.

In terms of ethnicity, Non-Hispanic Blacks, compared to other ethnic cohorts, have the highest prevalence of inadequate calcium and vitamin D intake and the lowest prevalence of calcium and vitamin D supplementation. Low income and poverty status increased the risk of these nutrient gaps, particularly among Non-Hispanic Black males. In contrast, Non-Hispanic Blacks are the least affected by osteoporosis when compared to other ethnic groups. Interestingly, low-income Non-Hispanic black males have a relative risk of osteoporosis two times greater than their higher income counterparts.

Burge et al. estimated an occurrence of more than 2 million incident fractures, with an economic burden to the US of $17 billion predicted to 2005 [4]. They predicted by 2025 that the incidence of fractures and their subsequent costs would rise roughly 50%, with an increase of more than 87% for people 65–74 years old and a 175% increase for Hispanics and individuals belonging to Other races.

The SNAP program is an effort to reduce food insecurity which affects one in seven households in the US [15]. Beneficiaries of the SNAP program includes children, people with disabilities and the elderly. There is evidence that SNAP benefit levels are not enough to last until the end of the month resulting in a 10–25% drop off in caloric intake by a month’s end. This is in turn has resulted in lower test scores and higher levels of disciplinary measures by month’s end among children receiving SNAP benefits.

Regarding the limitations of this analysis, the most obvious is that all differences in odds and risk tests are tests of association and not causation. It is expected that living in a state of poverty and being burdened by food insecurity is likely to lead to increased odds of being nutrient intake inadequate which can lead to increased risk of experiencing adverse events associated with metabolic-related diseases, like osteoporosis. This study provides statistically significant evidence of the directional association of these factors but more research is required to support more explicit statements of relation.

There is also a limitation in assessing a population that has osteoporosis as defined by bone health measure but may lack knowledge of their osteoporosis diagnosis. This analysis utilized an objective measurement to assess role of nutrient intake and sociodemographic factors on probable osteoporosis events as self-reported data on osteoporosis diagnosis may have induced bias. Also, while the NHANES sample obtained for this analysis for the total population is large enough to test for associations of the aforementioned factors by gender (n = 3,901), statistical testing for differences in risk of probable osteoporosis given poverty and food security states by ethnicity was constrained due to low number of observed cases of probable osteoporosis among some cohorts, such as male Non-Hispanic Blacks. It is likely that low number of observations observed in this cohort is naturally low as expected, but a larger sample size would only help to support this likely conclusion. Furthermore, the lack of standardization of ethnicity categorization across the different government data sources used in this analysis and especially how Hispanic people are categorized across the two platforms–NHANES and the US Census–adds to the complexity of determining the absolute size of the burden among the US population’s minority populations. This analysis only explored the associations of the odds of nutrient intake inadequacies and the risk of probable osteoporosis *across* various and mutually exclusive population cohorts. Thus, this analysis is cross-sectional in nature and temporal associations were not considered due to data limitations. Future research on this topic should explore the use of a longitudinal survey in order to explore the impact of changes in lifestyle behavior and changes in poverty status on osteoporosis risk factors.

## Supporting information

S1 Table Estimated population and sample sizes of key cohorts, United States, age 50 and older. (DOCX)

S2 Table Risk of osteoporosis, United States, by major population cohorts, %, age 50 and older. (DOCX)
