# Supplementary material for: Inadequate calcium and vitamin D intake and osteoporosis risk in older Americans living in poverty with food insecurities
Source: PLoS One. 2020 Jul 8;15(7):e0235042. doi: 10.1371/journal.pone.0235042 (PMC7343143; doi:10.1371/journal.pone.0235042)
Supplement: S1 Table — (DOCX) [file pone.0235042.s001.docx]

**S1 Table. Estimated population and sample sizes of key cohorts, united states, age 50 and older.**

| **Demographic Parameter** | | **Population** | **Inadequate Calcium Intake** | **Inadequate Vitamin D Intake** | **Calcium & Vitamin D Supplement Users** |
| --- | --- | --- | --- | --- | --- |
| **Female Cohort** | **Total Population** | 70.66 (100%) [1992] | 51.14 (72.4%) [1476] | 24.07 (34.1%) [760] | 17.01 (24.1%) [440] |
|  | ***Mexican American*** | [332] | [241] | [160] | [71] |
|  | ***Other Hispanic*** | [218] | [170] | [97] | [39] |
|  | ***Total Hispanic*** | 7.47 (10.6%) [550] | 5.58 (7.9%) [411] | 3.49 (4.9%) [257] | 1.49 (2.1%) [110] |
|  | ***Non-Hispanic Black*** | 7.87 (11.1%) [344] | 6.56 (9.3%) [287] | 3.98 (5.6%) [174] | 1.07 (1.5%) [47] |
|  | ***Non-Hispanic White*** | 50.55 (71.5%) [951] | 35.29 (49.9%) [664] | 15.2 (21.5%) [286] | 13.5 (19.1%) [254] |
|  | ***All Other People*** | 4.77 (6.8%) [147] | 3.7 (5.2%) [114] | 1.4 (2%) [43] | 0.94 (1.3%) [29] |
|  | **HH income below $20,000 per year** | 17.81 (25.2%) [524] | 13.24 (18.7%) [398] | 7.01 (9.9%) [221] | 4.13 (5.8%) [112] |
|  | ***Mexican American*** | [103] | [75] | [46] | [24] |
|  | ***Other Hispanic*** | [56] | [49] | [26] | [7] |
|  | ***Total Hispanic*** | 2.16 (3.1%) [159] | 1.68 (2.4%) [124] | 0.98 (1.4%) [72] | 0.42 (0.6%) [31] |
|  | ***Non-Hispanic Black*** | 2.33 (3.3%) [102] | 1.9 (2.7%) [83] | 1.3 (1.8%) [57] | 0.34 (0.5%) [15] |
|  | ***Non-Hispanic White*** | 12.28 (17.4%) [231] | 8.88 (12.6%) [167] | 4.46 (6.3%) [84] | 3.14 (4.4%) [59] |
|  | ***All Other People*** | 1.04 (1.5%) [32] | 0.78 (1.1%) [24] | 0.26 (0.4%) [8] | 0.23 (0.3%) [7] |
|  | **Monthly poverty level index less than or equal to 1.3** | 18.9 (26.7%) [602] | 14.48 (20.5%) [464] | 8.37 (11.8%) [279] | 3.78 (5.3%) [115] |
|  | ***Mexican American*** | [143] | [100] | [69] | [35] |
|  | ***Other Hispanic*** | [72] | [59] | [35] | [10] |
|  | ***Total Hispanic*** | 2.92 (4.1%) [215] | 2.16 (3.1%) [159] | 1.41 (2%) [104] | 0.61 (0.9%) [45] |
|  | ***Non-Hispanic Black*** | 2.77 (3.9%) [121] | 2.38 (3.4%) [104] | 1.56 (2.2%) [68] | 0.32 (0.5%) [14] |
|  | ***Non-Hispanic White*** | 11.75 (16.6%) [221] | 8.77 (12.4%) [165] | 4.94 (7%) [93] | 2.66 (3.8%) [50] |
|  | ***All Other People*** | 1.46 (2.1%) [45] | 1.17 (1.7%) [36] | 0.45 (0.6%) [14] | 0.19 (0.3%) [6] |
|  | **Food Insecure** | 8.43 (11.9%) [299] | 6.42 (9.1%) [228] | 4.11 (5.8%) [149] | 1.73 (2.4%) [57] |
|  | ***Mexican American*** | [74] | [50] | [36] | [18] |
|  | ***Other Hispanic*** | [53] | [43] | [31] | [5] |
|  | ***Total Hispanic*** | 1.72 (2.4%) [127] | 1.26 (1.8%) [93] | 0.91 (1.3%) [67] | 0.31 (0.4%) [23] |
|  | ***Non-Hispanic Black*** | 1.51 (2.1%) [66] | 1.26 (1.8%) [55] | 0.78 (1.1%) [34] | 0.23 (0.3%) [10] |
|  | ***Non-Hispanic White*** | 4.52 (6.4%) [85] | 3.35 (4.7%) [63] | 2.23 (3.2%) [42] | 1.06 (1.5%) [20] |
|  | ***All Other People*** | 0.68 (1%) [21] | 0.55 (0.8%) [17] | 0.19 (0.3%) [6] | 0.13 (0.2%) [4] |
|  | **SNAP Participants** | 8.68 (12.3%) [287] | 6.71 (9.5%) [222] | 4.15 (5.9%) [139] | 1.66 (2.3%) [55] |
|  | ***Mexican American*** | [61] | [40] | [32] | [17] |
|  | ***Other Hispanic*** | [39] | [30] | [16] | [8] |
|  | ***Total Hispanic*** | 1.36 (1.9%) [100] | 0.95 (1.3%) [70] | 0.65 (0.9%) [48] | 0.34 (0.5%) [25] |
|  | ***Non-Hispanic Black*** | 1.76 (2.5%) [77] | 1.58 (2.2%) [69] | 0.98 (1.4%) [43] | 0.16 (0.2%) [7] |
|  | ***Non-Hispanic White*** | 5.1 (7.2%) [96] | 3.83 (5.4%) [72] | 2.44 (3.5%) [46] | 1.06 (1.5%) [20] |
|  | ***All Other People*** | 0.45 (0.6%) [14] | 0.36 (0.5%) [11] | 0.06 (0.1%) [2] | 0.1 (0.1%) [3] |
| **Male Cohort** | **Total Population** | 62.02 (100%) [1909] | 32 (51.6%) [1035] | 24.63 (39.7%) [814] | 23.53 (37.9%) [685] |
|  | ***Mexican American*** | [316] | [168] | [143] | [120] |
|  | ***Other Hispanic*** | [195] | [120] | [95] | [57] |
|  | ***Total Hispanic*** | 6.67 (10.8%) [511] | 3.76 (6.1%) [288] | 3.11 (5%) [238] | 2.31 (3.7%) [177] |
|  | ***Non-Hispanic Black*** | 6.14 (9.9%) [363] | 3.91 (6.3%) [231] | 3.23 (5.2%) [191] | 1.71 (2.8%) [101] |
|  | ***Non-Hispanic White*** | 45.29 (73%) [899] | 22.12 (35.7%) [439] | 16.83 (27.1%) [334] | 18.19 (29.3%) [361] |
|  | ***All Other People*** | 3.91 (6.3%) [136] | 2.21 (3.6%) [77] | 1.47 (2.4%) [51] | 1.32 (2.1%) [46] |
|  | **HH income below $20,000 per year** | 12.74 (20.5%) [415] | 7.51 (12.1%) [258] | 5.95 (9.6%) [210] | 4.26 (6.9%) [127] |
|  | ***Mexican American*** | [71] | [43] | [40] | [23] |
|  | ***Other Hispanic*** | [44] | [28] | [21] | [12] |
|  | ***Total Hispanic*** | 1.5 (2.4%) [115] | 0.93 (1.5%) [71] | 0.8 (1.3%) [61] | 0.46 (0.7%) [35] |
|  | ***Non-Hispanic Black*** | 1.64 (2.6%) [97] | 1.27 (2%) [75] | 1.05 (1.7%) [62] | 0.29 (0.5%) [17] |
|  | ***Non-Hispanic White*** | 8.77 (14.1%) [174] | 4.89 (7.9%) [97] | 3.73 (6%) [74] | 3.17 (5.1%) [63] |
|  | ***All Other People*** | 0.83 (1.3%) [29] | 0.43 (0.7%) [15] | 0.37 (0.6%) [13] | 0.35 (0.6%) [12] |
|  | **Monthly poverty level index less than or equal to 1.3** | 15.25 (24.6%) [533] | 8.87 (14.3%) [322] | 7.55 (12.2%) [268] | 4.92 (7.9%) [166] |
|  | ***Mexican American*** | [115] | [63] | [51] | [44] |
|  | ***Other Hispanic*** | [67] | [43] | [35] | [18] |
|  | ***Total Hispanic*** | 2.38 (3.8%) [182] | 1.38 (2.2%) [106] | 1.12 (1.8%) [86] | 0.81 (1.3%) [62] |
|  | ***Non-Hispanic Black*** | 1.93 (3.1%) [114] | 1.42 (2.3%) [84] | 1.13 (1.8%) [67] | 0.41 (0.7%) [24] |
|  | ***Non-Hispanic White*** | 9.62 (15.5%) [191] | 5.29 (8.5%) [105] | 4.64 (7.5%) [92] | 3.27 (5.3%) [65] |
|  | ***All Other People*** | 1.32 (2.1%) [46] | 0.78 (1.3%) [27] | 0.66 (1.1%) [23] | 0.43 (0.7%) [15] |
|  | **Food Insecure** | 6.46 (10.4%) [265] | 3.77 (6.1%) [163] | 3.36 (5.4%) [140] | 2.03 (3.3%) [80] |
|  | ***Mexican American*** | [78] | [50] | [42] | [24] |
|  | ***Other Hispanic*** | [46] | [28] | [24] | [15] |
|  | ***Total Hispanic*** | 1.62 (2.6%) [124] | 1.02 (1.6%) [78] | 0.86 (1.4%) [66] | 0.51 (0.8%) [39] |
|  | ***Non-Hispanic Black*** | 0.98 (1.6%) [58] | 0.68 (1.1%) [40] | 0.56 (0.9%) [33] | 0.22 (0.4%) [13] |
|  | ***Non-Hispanic White*** | 3.43 (5.5%) [68] | 1.81 (2.9%) [36] | 1.76 (2.8%) [35] | 1.16 (1.9%) [23] |
|  | ***All Other People*** | 0.43 (0.7%) [15] | 0.26 (0.4%) [9] | 0.17 (0.3%) [6] | 0.14 (0.2%) [5] |
|  | **SNAP Participants** | 7.17 (11.6%) [266] | 3.68 (5.9%) [146] | 3.14 (5.1%) [122] | 2.88 (4.6%) [101] |
|  | ***Mexican American*** | [43] | [19] | [21] | [19] |
|  | ***Other Hispanic*** | [38] | [23] | [16] | [15] |
|  | ***Total Hispanic*** | 1.06 (1.7%) [81] | 0.55 (0.9%) [42] | 0.48 (0.8%) [37] | 0.44 (0.7%) [34] |
|  | ***Non-Hispanic Black*** | 1.34 (2.2%) [79] | 0.91 (1.5%) [54] | 0.71 (1.1%) [42] | 0.36 (0.6%) [21] |
|  | ***Non-Hispanic White*** | 4.03 (6.5%) [80] | 1.81 (2.9%) [36] | 1.66 (2.7%) [33] | 1.76 (2.8%) [35] |
|  | ***All Other People*** | 0.75 (1.2%) [26] | 0.4 (0.6%) [14] | 0.29 (0.5%) [10] | 0.32 (0.5%) [11] |
| **All Cohort** | **Total Population** | 132.68 (100%) [3901] | 83.14 (62.7%) [2511] | 48.7 (36.7%) [1574] | 40.54 (30.6%) [1125] |
|  | ***Mexican American*** | [648] | [409] | [303] | [191] |
|  | ***Other Hispanic*** | [413] | [290] | [192] | [96] |
|  | ***Total Hispanic*** | 14.14 (10.7%) [1061] | 9.34 (7%) [699] | 6.6 (5%) [495] | 3.8 (2.9%) [287] |
|  | ***Non-Hispanic Black*** | 14.01 (10.6%) [707] | 10.47 (7.9%) [518] | 7.21 (5.4%) [365] | 2.78 (2.1%) [148] |
|  | ***Non-Hispanic White*** | 95.84 (72.2%) [1850] | 57.41 (43.3%) [1103] | 32.03 (24.1%) [620] | 31.69 (23.9%) [615] |
|  | ***All Other People*** | 8.69 (6.5%) [283] | 5.92 (4.5%) [191] | 2.86 (2.2%) [94] | 2.26 (1.7%) [75] |
|  | **HH income below $20,000 per year** | 30.55 (23%) [939] | 20.75 (15.6%) [656] | 12.95 (9.8%) [431] | 8.39 (6.3%) [239] |
|  | ***Mexican American*** | [174] | [118] | [86] | [47] |
|  | ***Other Hispanic*** | [100] | [77] | [47] | [19] |
|  | ***Total Hispanic*** | 3.66 (2.8%) [274] | 2.61 (2%) [195] | 1.77 (1.3%) [133] | 0.88 (0.7%) [66] |
|  | ***Non-Hispanic Black*** | 3.97 (3%) [199] | 3.17 (2.4%) [158] | 2.35 (1.8%) [119] | 0.63 (0.5%) [32] |
|  | ***Non-Hispanic White*** | 21.04 (15.9%) [405] | 13.76 (10.4%) [264] | 8.19 (6.2%) [158] | 6.31 (4.8%) [122] |
|  | ***All Other People*** | 1.87 (1.4%) [61] | 1.21 (0.9%) [39] | 0.63 (0.5%) [21] | 0.57 (0.4%) [19] |
|  | **Monthly poverty level index less than or equal to 1.3** | 34.15 (25.7%) [1135] | 23.35 (17.6%) [786] | 15.92 (12%) [547] | 8.71 (6.6%) [281] |
|  | ***Mexican American*** | [258] | [163] | [120] | [79] |
|  | ***Other Hispanic*** | [139] | [102] | [70] | [28] |
|  | ***Total Hispanic*** | 5.3 (4%) [397] | 3.54 (2.7%) [265] | 2.53 (1.9%) [190] | 1.42 (1.1%) [107] |
|  | ***Non-Hispanic Black*** | 4.7 (3.5%) [235] | 3.8 (2.9%) [188] | 2.69 (2%) [135] | 0.73 (0.6%) [38] |
|  | ***Non-Hispanic White*** | 21.37 (16.1%) [412] | 14.06 (10.6%) [270] | 9.58 (7.2%) [185] | 5.93 (4.5%) [115] |
|  | ***All Other People*** | 2.78 (2.1%) [91] | 1.95 (1.5%) [63] | 1.12 (0.8%) [37] | 0.63 (0.5%) [21] |
|  | **Food Insecure** | 14.89 (11.2%) [564] | 10.19 (7.7%) [391] | 7.47 (5.6%) [289] | 3.77 (2.8%) [137] |
|  | ***Mexican American*** | [152] | [100] | [78] | [42] |
|  | ***Other Hispanic*** | [99] | [71] | [55] | [20] |
|  | ***Total Hispanic*** | 3.34 (2.5%) [251] | 2.28 (1.7%) [171] | 1.77 (1.3%) [133] | 0.82 (0.6%) [62] |
|  | ***Non-Hispanic Black*** | 2.49 (1.9%) [124] | 1.93 (1.5%) [95] | 1.34 (1%) [67] | 0.45 (0.3%) [23] |
|  | ***Non-Hispanic White*** | 7.94 (6%) [153] | 5.16 (3.9%) [99] | 4 (3%) [77] | 2.22 (1.7%) [43] |
|  | ***All Other People*** | 1.11 (0.8%) [36] | 0.81 (0.6%) [26] | 0.37 (0.3%) [12] | 0.27 (0.2%) [9] |
|  | **SNAP Participants** | 15.85 (11.9%) [553] | 10.39 (7.8%) [368] | 7.29 (5.5%) [261] | 4.54 (3.4%) [156] |
|  | ***Mexican American*** | [104] | [59] | [53] | [36] |
|  | ***Other Hispanic*** | [77] | [53] | [32] | [23] |
|  | ***Total Hispanic*** | 2.42 (1.8%) [181] | 1.5 (1.1%) [112] | 1.13 (0.9%) [85] | 0.78 (0.6%) [59] |
|  | ***Non-Hispanic Black*** | 3.1 (2.3%) [156] | 2.49 (1.9%) [123] | 1.69 (1.3%) [85] | 0.52 (0.4%) [28] |
|  | ***Non-Hispanic White*** | 9.13 (6.9%) [176] | 5.64 (4.3%) [108] | 4.11 (3.1%) [79] | 2.83 (2.1%) [55] |
|  | ***All Other People*** | 1.2 (0.9%) [40] | 0.76 (0.6%) [25] | 0.35 (0.3%) [12] | 0.41 (0.3%) [14] |

Total population in millions of people. Share of estimated population in parentheses. Sample Size in brackets.
