# Supplementary material for: Inadequate calcium and vitamin D intake and osteoporosis risk in older Americans living in poverty with food insecurities
Source: PLoS One. 2020 Jul 8;15(7):e0235042. doi: 10.1371/journal.pone.0235042 (PMC7343143; doi:10.1371/journal.pone.0235042)
Supplement: S2 Table — (DOCX) [file pone.0235042.s002.docx]

**S2 Table. Risk of osteoporosis, united states, by major population cohorts, %, age 50 and older.**

| **Demographic Parameter** | | **Population** | **Inadequate Calcium Intake** | **Inadequate Vitamin D Intake** | **Calcium & Vitamin D Supplement Users** |  |
| --- | --- | --- | --- | --- | --- | --- |
| **Female Cohort** | **Total Population** | 16.2 (0.01) | 16.3 (0.01) | 16.6 (0.02) | 16.4 (0.03) |  |
|  | ***Mexican American*** | 19.3 (0.02) | 19.5 (0.03) | 22.5 (0.05) | 22.5 (0.09) |  |
|  | ***Other Hispanic*** | 21.6 (0.03) | 24.1 (0.04) | 20.6 (0.07) | 15.4 (0.11) |  |
|  | ***Non-Hispanic Black*** | 8.4 (0.02) | 8.7 (0.02) | 6.9 (0.01) | 4.3 (0.06) |  |
|  | ***Non-Hispanic White*** | 15 (0.01) | 15.1 (0.01) | 17.8 (0.03) | 14.6 (0.04) |  |
|  | ***All Other People*** | 27.2 (0.05) | 24.6 (0.05) | 16.3 (0.09) | 37.9 (0.17) |  |
|  | **HH income below $20,000 per year** | 21.6 (0.04) | 21.1 (0.04) | 19.5 (0.05) | 24.1 (0.08) |  |
|  | ***Mexican American*** | 25.2 (0.08) | 26.7 (0.1) | 34.8 (0.13) | 25 (0.18) |  |
|  | ***Other Hispanic*** | 26.8 (0.12) | 26.5 (0.13) | 19.2 (0.16) | 28.6 (0.44) |  |
|  | ***Non-Hispanic Black*** | 7.8 (0.05) | 9.6 (0.07) | 5.3 (0.01) | -- |  |
|  | ***Non-Hispanic White*** | 22.9 (0.05) | 21.6 (0.06) | 22.6 (0.09) | 25.4 (0.11) |  |
|  | ***All Other People*** | 34.4 (0.16) | 29.2 (0.19) | -- | 57.1 (0.4) |  |
|  | **Monthly poverty level index less than or equal to 1.3** | 17.8 (0.03) | 16.4 (0.04) | 15.4 (0.04) | 24.3 (0.08) |  |
|  | ***Mexican American*** | 23.1 (0.07) | 23 (0.09) | 26.1 (0.1) | 28.6 (0.15) |  |
|  | ***Other Hispanic*** | 19.4 (0.1) | 18.6 (0.11) | 11.4 (0.12) | 30 (0.34) |  |
|  | ***Non-Hispanic Black*** | 6.6 (0.04) | 7.7 (0.05) | 2.9 (0.08) | -- |  |
|  | ***Non-Hispanic White*** | 17.6 (0.05) | 14.5 (0.06) | 19.4 (0.08) | 24 (0.12) |  |
|  | ***All Other People*** | 28.9 (0.13) | 27.8 (0.15) | -- | 50 (0.46) |  |
|  | **Food Insecure** | 17.4 (0.04) | 15.8 (0.05) | 13.4 (0.04) | 26.3 (0.11) |  |
|  | ***Mexican American*** | 23 (0.1) | 20 (0.11) | 22.2 (0.12) | 38.9 (0.22) |  |
|  | ***Other Hispanic*** | 15.1 (0.1) | 16.3 (0.12) | 6.5 (0.04) | -- |  |
|  | ***Non-Hispanic Black*** | 6.1 (0.03) | 7.3 (0.06) | -- | -- |  |
|  | ***Non-Hispanic White*** | 22.4 (0.09) | 20.6 (0.1) | 21.4 (0.11) | 25 (0.2) |  |
|  | ***All Other People*** | 19 (0.19) | 11.8 (0.21) | -- | 50 (0.63) |  |
|  | **SNAP Participants** | 15.3 (0.04) | 16.2 (0.04) | 16.5 (0.05) | 9.1 (0.03) |  |
|  | ***Mexican American*** | 21.3 (0.09) | 30 (0.13) | 31.3 (0.14) | -- |  |
|  | ***Other Hispanic*** | 7.7 (0.04) | 6.7 (0.02) | -- | -- |  |
|  | ***Non-Hispanic Black*** | 9.1 (0.04) | 10.1 (0.05) | -- | -- |  |
|  | ***Non-Hispanic White*** | 19.8 (0.07) | 19.4 (0.08) | 23.9 (0.1) | 10 (0.1) |  |
|  | ***All Other People*** | 14.3 (0.21) | -- | -- | -- |  |
| **Male Cohort** | **Total Population** | 4.6 (0.03) | 5.2 (0.04) | 4.3 (0.03) | 3.9 (0.02) |  |
|  | ***Mexican American*** | 3.8 (0.12) | 4.2 (0.14) | 4.9 (0.05) | 2.5 (0.13) |  |
|  | ***Other Hispanic*** | 4.6 (0.11) | 2.5 (0.39) | -- | 10.5 (0.07) |  |
|  | ***Non-Hispanic Black*** | 1.9 (0.28) | 2.6 (0.24) | 2.6 (0.15) | -- |  |
|  | ***Non-Hispanic White*** | 5.2 (0.04) | 7.3 (0.03) | 5.1 (0.03) | 3.3 (0.04) |  |
|  | ***All Other People*** | 8.8 (0.03) | 7.8 (0.06) | 9.8 (0.04) | 10.9 (0.08) |  |
|  | **HH income below $20,000 per year** | 6.3 (0.02) | 6.2 (0.02) | 6.2 (0.01) | 6.3 (0.02) |  |
|  | ***Mexican American*** | 4.2 (0) | 4.7 (0.02) | -- | -- |  |
|  | ***Other Hispanic*** | 4.5 (0.02) | -- | -- | 16.7 (0.29) |  |
|  | ***Non-Hispanic Black*** | 3.1 (0.05) | 4 (0.01) | 4.8 (0.01) | -- |  |
|  | ***Non-Hispanic White*** | 8 (0.04) | 9.3 (0.06) | 6.8 (0.03) | 6.3 (0.03) |  |
|  | ***All Other People*** | 13.8 (0.15) | 13.3 (0.25) | 23.1 (0.27) | -- |  |
|  | **Monthly poverty level index less than or equal to 1.3** | 6.8 (0.02) | 8.1 (0.03) | 7.5 (0.03) | 4.2 (0.01) |  |
|  | ***Mexican American*** | 3.5 (0.01) | 4.8 (0.03) | 5.9 (0.05) | -- |  |
|  | ***Other Hispanic*** | 4.5 (0.02) | -- | -- | 11.1 (0.22) |  |
|  | ***Non-Hispanic Black*** | 4.4 (0.01) | 6 (0.04) | 6 (0.04) | -- |  |
|  | ***Non-Hispanic White*** | 9.9 (0.05) | 13.3 (0.07) | 9.8 (0.06) | 6.2 (0.05) |  |
|  | ***All Other People*** | 10.9 (0.11) | 11.1 (0.16) | 17.4 (0.18) | -- |  |
|  | **Food Insecure** | 4.2 (0.01) | 3.7 (0.02) | 3.6 (0.1) | 3.8 (0.06) |  |
|  | ***Mexican American*** | -- | -- | -- | -- |  |
|  | ***Other Hispanic*** | 4.3 (0.02) | -- | -- | 13.3 (0.21) |  |
|  | ***Non-Hispanic Black*** | -- | -- | -- | -- |  |
|  | ***Non-Hispanic White*** | 8.8 (0.06) | 11.1 (0.11) | 5.7 (0.07) | -- |  |
|  | ***All Other People*** | 13.3 (0.24) | -- | 33.3 (0.45) | -- |  |
|  | **SNAP Participants** | 7.9 (0.01) | 7.5 (0.02) | 9 (0.01) | 6.9 (0) |  |
|  | ***Mexican American*** | -- | -- | -- | -- |  |
|  | ***Other Hispanic*** | 13.2 (0.1) | -- | -- | 26.7 (0.22) |  |
|  | ***Non-Hispanic Black*** | 5.1 (0.03) | 7.4 (0.03) | 9.5 (0.03) | -- |  |
|  | ***Non-Hispanic White*** | 10 (0.05) | 11.1 (0.09) | 9.1 (0.03) | 8.6 (0.04) |  |
|  | ***All Other People*** | 11.5 (0.11) | 14.3 (0.21) | 30 (0.29) | -- |  |
| **All Cohort** | **Total Population** | 10.5 (0.01) | 11.7 (0.01) | 10.2 (0.01) | 8.8 (0.01) |  |
|  | ***Mexican American*** | 11.7 (0.02) | 13.2 (0.03) | 14.2 (0.03) | 9.9 (0.03) |  |
|  | ***Other Hispanic*** | 13.6 (0.03) | 15.2 (0.04) | 10.9 (0.02) | 12.5 (0.06) |  |
|  | ***Non-Hispanic Black*** | 5.1 (0.00) | 6 (0.01) | 4.7 (0.03) | 2 (0.17) |  |
|  | ***Non-Hispanic White*** | 10.3 (0.01) | 12 (0.02) | 11 (0.01) | 8 (0.01) |  |
|  | ***All Other People*** | 18.4 (0.04) | 17.8 (0.05) | 12.8 (0.04) | 21.3 (0.09) |  |
|  | **HH income below $20,000 per year** | 14.8 (0.02) | 15.2 (0.03) | 13 (0.03) | 14.6 (0.04) |  |
|  | ***Mexican American*** | 16.7 (0.06) | 18.6 (0.07) | 20.9 (0.08) | 14.9 (0.1) |  |
|  | ***Other Hispanic*** | 17 (0.08) | 16.9 (0.09) | 10.6 (0.09) | 21.1 (0.2) |  |
|  | ***Non-Hispanic Black*** | 5.5 (0.02) | 7 (0.03) | 5 (0.00) | -- |  |
|  | ***Non-Hispanic White*** | 16.5 (0.04) | 17 (0.05) | 15.2 (0.05) | 15.6 (0.06) |  |
|  | ***All Other People*** | 24.6 (0.11) | 23.1 (0.14) | 14.3 (0.17) | 26.3 (0.21) |  |
|  | **Monthly poverty level index less than or equal to 1.3** | 12.6 (0.02) | 13 (0.03) | 11.5 (0.03) | 12.5 (0.04) |  |
|  | ***Mexican American*** | 14.3 (0.05) | 16 (0.06) | 17.5 (0.07) | 12.7 (0.08) |  |
|  | ***Other Hispanic*** | 12.2 (0.06) | 11.8 (0.07) | 5.7 (0.03) | 17.9 (0.17) |  |
|  | ***Non-Hispanic Black*** | 5.5 (0.02) | 6.9 (0.03) | 4.4 (0.00) | -- |  |
|  | ***Non-Hispanic White*** | 14.1 (0.04) | 14.1 (0.05) | 14.6 (0.05) | 13.9 (0.07) |  |
|  | ***All Other People*** | 19.8 (0.09) | 20.6 (0.11) | 13.5 (0.13) | 19 (0.2) |  |
|  | **Food Insecure** | 11.2 (0.02) | 10.7 (0.03) | 8.7 (0.01) | 13.1 (0.05) |  |
|  | ***Mexican American*** | 11.2 (0.05) | 10 (0.06) | 10.3 (0.03) | 16.7 (0.11) |  |
|  | ***Other Hispanic*** | 10.1 (0.06) | 9.9 (0.07) | 3.6 (0.2) | 15 (0.17) |  |
|  | ***Non-Hispanic Black*** | 4 (0.02) | 5.3 (0.01) | 3 (0.26) | -- |  |
|  | ***Non-Hispanic White*** | 16.3 (0.06) | 17.2 (0.08) | 14.3 (0.06) | 14 (0.1) |  |
|  | ***All Other People*** | 16.7 (0.13) | 11.5 (0.14) | 16.7 (0.23) | 22.2 (0.35) |  |
|  | **SNAP Participants** | 11.8 (0.02) | 12.8 (0.03) | 13 (0.03) | 7.7 (0.01) |  |
|  | ***Mexican American*** | 13.5 (0.05) | 20.3 (0.09) | 20.8 (0.09) | -- |  |
|  | ***Other Hispanic*** | 10.4 (0.05) | 5.7 (0.02) | -- | 21.7 (0.16) |  |
|  | ***Non-Hispanic Black*** | 7.1 (0.01) | 8.9 (0.03) | 5.9 (0.04) | -- |  |
|  | ***Non-Hispanic White*** | 15.3 (0.05) | 16.7 (0.06) | 17.7 (0.07) | 9.1 (0.03) |  |
|  | ***All Other People*** | 12.5 (0.09) | 12 (0.12) | 25 (0.24) | -- |  |

Standard Errors are in Parentheses. “--“ indicates insufficient sample sizes for statistical analysis.
